# Neuropathy-associated Tecpr2 mutation knock-in mice reveal endolysosomal loss of function phenotypes in neurons and microglia

**DOI:** 10.1038/s41419-025-08168-w

**Published:** 2025-10-31

**Authors:** Debjani Bhattacharya, Patricia da Silva-Buttkus, Karsten Nalbach, Lizhen Cheng, Lillian Garrett, Martin Irmler, Georg Kislinger, Georg Werner, Ramona Rodde, Christoph Lengger, Johannes Beckers, Annemarie Zimprich, Sabine M. Hölter, Valerie Gailus-Durner, Helmut Fuchs, Martin Hrabe de Angelis, Benedikt Wefers, Wolfgang Wurst, Monika S. Brill, Martina Schifferer, Stefan F. Lichtenthaler, Christian Behrends

**Affiliations:** 1 Munich Cluster for Systems Neurology (SyNergy), Faculty of Medicine, Ludwig-Maximilians-Universität München, Munich, Germany; 2 Institute of Experimental Genetics and German Mouse Clinic, Helmholtz Zentrum München, German Research Center for Environmental Health, Neuherberg, Germany; 3 German Center for Neurodegenerative Diseases (DZNE), Munich, Germany; 4 Neuroproteomics, School of Medicine and Health, Klinikum rechts der Isar, Technical University of Munich, Munich, Germany; 5 Institute of Neuronal Cell Biology, Technical University of Munich, Munich, Germany; 6 Metabolic Biochemistry, Biomedical Center (BMC), Faculty of Medicine, Ludwig- Maximilians-Universität München, Munich, Germany; 7 German Center for Mental Health (DZPG), Munich site, Germany; 8 Chair of Experimental Genetics, TUM School of Life Sciences, Technische Universität München, Freising, Germany; 9 German Center for Diabetes Research (DZD), Neuherberg, Germany; 10 Institute of Developmental Genetics, Helmholtz Zentrum München, Munich, Germany; 11 Munich Cluster for Systems Neurology (SyNergy), Munich, Germany

**Keywords:** Lysosomes, Neurodegeneration

## Abstract

Mutations in the gene encoding Tectonic β-propeller repeat-containing repeat protein 2 (TECPR2) cause hereditary sensory and autonomic neuropathy subtype 9 (HSAN9) which is a fatal neurodevelopmental and neurodegenerative disorder involving the sensory and peripheral nervous system. TECPR2 is ubiquitously expressed and linked to trafficking and sorting within the cell, however, its functional role remains poorly defined. Moreover, molecular insights into pathogenic mechanisms underlying HSAN9 are lacking. Here, we report a novel mouse model which harbors a HSAN9-associated nonsense mutation that causes loss of TECPR2 expression. Mice show altered gait, highly region-specific axonal dystrophy, and extensive local gliosis. The affected medulla area prominently features swollen axons filled with amorphous protein aggregates, glycogen granules, single and double membrane vesicles as well as aberrant organelles including ER and mitochondria whose proteome is distinctly altered. Despite the locally restricted pathology the neuronal demise is detectable in the cerebrospinal fluid and responded to by damage-associated microglia. However, their capacity to clear neuronal debris seems attenuated. Overall, neuronal and microglia phenotypes point to a dysfunctional endolysosomal system when TECPR2 is missing. This was confirmed in TECPR2 knockout cells and linked to TECPR2’s interaction with the homotypic fusion and protein sorting (HOPS)–tethering complex. Collectively, we uncovered a role of TECPR2 in endolysosome maintenance which seems relevant for healthy neurons in a particular brain region.

## Introduction

Hereditary sensory and autonomic neuropathy (HSAN) is a group of fatal genetic diseases involving a varied array of neurological disorders. It is characterized by axonal dystrophy and degeneration affecting sensory as well as peripheral nervous system involving control of respiration, gastrointestinal reflux and lower limb movements [1]. HSAN subtype 9 (HSAN9) is an autosomal recessive neurodevelopmental and neurodegenerative disorder caused due to homozygous or compound heterozygous mutations in a gene encoding Tectonic β-propeller repeat-containing repeat protein 2 (TECPR2). HSAN9 patients display an overall developmental delay and intellectual disability. They also develop muscle spasticity, hyporeflexia which manifests as walking disability, gait ataxia and often progress to hypoventilation, impaired blood pressure regulation, and frequent aspiration events. Early mortality in these patients is primarily due to dysregulation in respiratory control [2–5].

TECPR2 is comprised of N-terminal tryptophan aspartic acid 40 (WD40) repeats and Tectonic β propeller repeats (TECPRs) along with an LC3-interacting region (LIR) at the C-terminus. TECPR2 was first identified as an ATG8-interacting protein and shown to be a positive regulator of autophagy [2, 6]. TECPR2 also interacts with SEC24D, an endoplasmic reticulum (ER) protein involved in the formation and maintenance of COPII vesicles and ER exit site (ERES). Loss of TECPR2 destabilizes ER-to-Golgi transport through a reduction of ERES [7]. Besides, TECPR2 also interacts with several subunits belonging to protein complexes such as biogenesis of lysosome-related organelle complex 1 (BLOC1) and homotypic fusion and protein sorting (HOPS). BLOC1 plays various roles in vesicle biogenesis, cargo sorting, and vesicle recycling whereas HOPS functions in vesicle tethering and fusion of late endosomes and autophagosomes with lysosomes [8, 9].

Nonsense mutations such as p.Leu440Argfs*19 (L440Rfs) or p.Leu1139Argfs*75 (L1139Rfs) are thought to yield premature stop codons which in turn result in partial or complete functional loss of TECPR2. Consistently, fibroblasts from HSAN9 patients carrying the L440Rfs mutation show no detectable TECPR2 expression and are characterized by accumulation of autophagosomes and loss of TECPR2’s interaction with the lysosomal SNARE protein VAMP8, indicating a role of TECPR2 in vesicle tethering and fusion [10]. Likewise, CRISPR/Cas9-engineering of L440Rfs in HeLa cells yielded no TECPR2 expression. Spatial and proximity proteomics of these cells unveiled profound changes in the composition of ERES, COPII carriers and lysosomes as well as altered proteomes of the plasma membrane and the secreted media. Intriguingly, changes in the latter two sub-proteomes could not be rescued by overexpressing TECPR2 L440Rfs whose interactions with trafficking components such as HOPS was lost or substantially reduced [11]. Hence, the functional consequences of compromised TECPR2 expression are not restricted to defective ER-to-Golgi transport but expand to other cellular compartments which can be reached via the secretory pathway. Given TECPR2’s interaction with HOPS, some of these sorting defects might not be related to the role of TECPR2 in the early secretory pathway but could rather reflect a function in the endolysosomal system. Consistent with this notion, increased LAMP1-positive structures and decreased lysosomal mTORC1 were not only observed in cells lacking TECPR2 but also reported upon deficiency of the HOPS subunit VPS41 [10–12]. However, the molecular basis for these endolysosomal abnormalities and the functional significance of TECPR2’s interaction with HOPS remain largely elusive.

*Tecpr2* knock-out (KO) mice show motor and sensory neurodegeneration which is consistent with some of the clinical manifestations in HSAN9 patients [13]. Neuropathologically, these mice featured neuronal degeneration in specific mid- and hindbrain assessed by MRI accompanied by decreased neurofilaments in the spinal cord as well as axonal swelling and autophagosome accumulation in the medulla. Importantly, the latter two worsened in an age-dependent manner [13]. However, *Tecpr2* KO mice do not faithfully recapitulate disease pathology induced by HSAN9-associated mutations since possible toxic gain-of-functions of TECPR2 mutant variants cannot be assessed in this model. In addition, the characterization of Tecpr2 KO mice focused on neurons while *TECPR2* mRNA is ubiquitously expressed. Hence, the roles of different brain cell types involved in HSAN9 pathogenesis are yet to be deciphered.

To overcome these limitations, we generated a *Tecpr2* knock-in (ki/ki) mouse model mimicking the nonsense mutations L1139Rfs. While this frameshift mutation is in exon 16 encoding parts of TECPRs and could therefore produce a C-terminal truncated mutant variant, TECPR2 was not detected at the protein level and its mRNA was significantly decreased. Behavioral and phenotypic studies of these mice showed a gait ataxia phenotype as observed in HSAN9 patients and axonal dystrophy as observed in *Tecpr2* KO mice. Intriguingly, the presence of axonal swelling in a specific region of the medulla correlated with neuronal loss in this area. Proteomics and microscopic analysis of the affected medulla region revealed altered organelle and protein homeostasis characterized by accumulation of amorphous protein aggregates, glycogen granules, autophagosomes and synaptic vesicles as well as enlarged ER and swollen mitochondria whose proteomes were also altered. Collectively, these alterations point to a defective endolysosomal system in medulla neurons. Consistent with prominent gliosis, transcriptomics of *Tecpr2* ki/ki mouse brains unveiled an increase in disease-associated microglia (DAM) genes. The DAM state was confirmed by immunohistochemistry along with microglia proteomics and immunofluorescence which showed increased lysosomal proteins and a decrease in endocytic processes. To better understand the impact of TECPR2 on the endolysosomal system in neurons and microglia, we turned to TECPR2 KO cells and characterized TECPR2’s binding to HOPS. We concluded that loss of TECPR2 causes an overall alteration of the endolysosomal system which possibly leads to the accumulation of cellular components destined for lysosomal degradation in neurons and defective clearing of endocytic and phagocytic cargo in microglia. We speculate that axonal dystrophy in the medulla likely triggers the activation of microglia whose endolysosomal system is also functionally compromised by the lack of TECPR2. This dysfunctional neuron-glia interaction might contribute to HSAN9-related phenotypes in *Tecpr2* ki/ki mice and possibly in HSAN9 patients.

## Methods

### Creation of Tecpr2^L1152Yfs^ mice

Tecpr2^L1152Yfs^ mice were generated using CRISPR/Cas9-mediated gene editing in zygotes, as previously outlined. [14] In short, pronuclear stage zygotes were obtained by mating C57BL/6 J male mice with superovulated C57BL/6 J female mice. Embryos were then electroporated using the NEPA21 electroporator and a 1 mm electrode with a Tecpr2-specific CRISPR/Cas9 ribonucleoprotein (RNP) solution consisting of 200 ng/μl S.p. Cas9 WT protein (IDT), 6 μM crRNA (protospacer GCTGCTCTGGCATAGCCATA; IDT), 6 μM tracrRNA (IDT), and 300 ng/μl mutagenic single-stranded oligodeoxynucleotide (ssODN), carrying a 1 bp deletion (ΔT, L1152Yfs) and a silent mutation (TCC > TCA) for genotyping purposes. Post-electroporation, zygotes were implanted into pseudopregnant CD-1 surrogate animals. Mutant founder animals carrying the L1152Yfs allele were identified and then crossed to C57BL/6 J animals. Validation of the targeted locus was performed on genomic DNA from F1 animals by RFLP and Sanger sequencing. Potential off-target sites for the Tecpr2-specific crRNA were predicted using the CRISPR online tool [15] to eliminate any unwanted modifications. Genomic DNA from the F1 generation was PCR-amplified and confirmed through Sanger sequencing, revealing no additional sequence variations. All animal experiments were approved by the Bavarian government under license number ROB-55.2Vet-2532.Vet_02-16-121. Mouse phenotyping was performed at the German Mouse Clinic (GMC) [16, 17] under license number ROB-55.2-2532.Vet_02-20-202. All mice were treated in compliance with the institutional guidelines approved by the animal welfare and use committee of the government of Upper Bavaria. They were housed in standard cages in a specific pathogen-free facility, maintained on a 12-h light/dark cycle, and given unrestricted access to food and water. The sample size of 15 mice per sex and genotype based on phenotyping experience at the GMC was sufficient to detect medium differences of one standard deviation with a power of 0.8 and an alpha level of 0.05. Deviations from this number are described. Mice were selected from a list and identities were assigned to the experiment. Female and male mice were housed separately, while genotypes were mixed within cages. Phenotypic data, excluding histopathological assessments, were automatically recorded with metadata stored concurrently.

### Open field (OF) test

The open field test (9 weeks of age, *n* = 15/sex/genotype,) was carried out as described previously [18, 19]. The test apparatus from ActiMot, TSE was a square-shaped frame with two pairs of light-beam strips, each pair consisting of one transmitter strip and one receiver strip. These basic light barrier strips were arranged at right angles to each other in the same plane to determine the X and Y coordinates of the animal, and thus its location (XY frame). Each strip was equipped with 16 infrared sensors with a distance between adjacent sensors of 28 mm. With two further pairs of uni-dimensional light-barrier strips (Z1 and Z2), rearing could be detected in addition to location. The light barriers were scanned with a frequency of 100 Hz each on fast computer platforms. The test apparatus consisted of a transparent and infrared light permeable acrylic test arena (internal measurements: 45.5 × 45.5 × 39.5 cm) with a smooth floor. The illumination levels were set at approximately 150 lux in the corners and 200 lux in the middle of the test arena.

### Prepulse inhibition

For prepulse inhibition (PPI) of the acoustic startle reflex (ASR) (10 weeks of age, n = 15/sex/ genotype), we applied a method we described previously [20]. The ASR/PPI assesses two functions: sensorimotor recruitment (ASR) and sensorimotor gating (PPI). The latter reflects the ability to filter irrelevant sensory information. The experimental apparatus consisted of an outer sound-attenuated chamber and an inner load cell platform that recorded the startle response (Med Associates Inc.). After a 5-min acclimation period, the startle response to a 110 dB pulse was measured. For PPI assessment, the capacity of a weaker acoustic pre-pulse (from 67, 69, 73 to 81 dB) to attenuate a 110 dB pulse was calculated as a percentage score. For each acoustic pre-pulse trial, % PPI = 100 x (S – PPi_S)/S, where S is the basal startle response at 110 dB and PPI is the startle response after the pre-pulse exposure. The protocol consisted of 10 blocks, each including every trial condition. ASR was also elicited 10 times at 110 dB without a pre-pulse to determine the baseline response.

### CatWalk

Animals (10 male wt/wt, 12 female wt/wt, 12 male ki/ki, 12 female ki/ki) were tested in Catwalk (CW) XT® system (Noldus Information Technology, Wageningen, Netherlands) [21] from the age of 19-22 weeks (about 5 months). In this system, the mouse traverses an elevated glass walkway bordered by Plexiglas walls in a dark room. A camera beneath the walkway tracks the illuminated footprints that are analyzed with the CW software. For each animal the mean of 2–4 continuous runs (each included approx. 4–6 step cycles) were calculated and used for the analysis. The high-speed camera captured the footprints with a high-speed camera at a rate of up to 100 Hz. The CW system measures aspects of paw floor contact dynamics and calculates many spatial and temporal gait parameters. These include (i) individual paw print parameters, such as the width and length, with a calculation for the front and hind paws (FP and HP, respectively); (ii) paw print position with respect to each other parameters, such as the stride length and the base of support (BOS), indexing the width between the two FPs and the two HPs respectively; (iii) parameters related to time-based relationships between paw pairs (couplings and phase dispersion) and their variation.

### Statistics for behavior studies

Statistical analysis was performed using SPSS® and Graphpad Prism version 8.0.2. All parameters were analyzed using 2-way ANOVA with sex and genotype as factors. When no significant sex x genotype interaction effect was detected, the data of males and females was pooled for all graphs depicted. When an interaction effect was detected, the males and females were graphed separately and a post-hoc Bonferroni’s test was performed. The significance threshold was *p* < 0.05.

### Histological and immunohistological analyses of brain tissue and spinal cord

Tissues collected from wt/wt and ki/ki mice (23 weeks of age, *n* = 5/sex/genotype or 8 weeks of age, *n* = 1/sex/genotype) were fixed in neutral buffered formalin, processed, embedded in paraffin, sectioned at 3 µm and stained with standard hematoxylin and eosin for high-throughput blind histopathological evaluation as previously described [17]. Brain sections were further examined with Periodic-Acid-Schiff (PAS), PAS-Diastase and Congo Red. The immunohistochemical detections were performed using a BOND RX^m^ (Leica Biosystems, Germany) automated stainer. Brain sections were deparaffinized, antigens were retrieved with citrate buffer (Bond Epitope Retrieval Solution 1-H1) or ethylenediaminetetraacetic acid (EDTA) (Bond Epitope Retrieval Solution 2-H2) for 30 min and blocked for 30 min with blocking agent. Primary antibodies (Supplementary Table 8) were applied and incubated for 60 min followed by secondary antibodies applied for 30 min. The detection was performed with BOND Polymer Refine Detection DAB (3,3’-diaminobenzidine). Slides were counterstained with hematoxylin, imaged with a NanoZoomer S60 scanner (Hamamatsu, Japan) and examined with NDP.View2 Plus Software. In negative-control sections, the primary antibodies were omitted and antibody diluent was used. The quantification of the number CTIP2-positive neuron cells in the brain cortex (*n* = 5/genotype/sex) was performed using the open-source software QuPath version 0.5.1 [22]. The region of interest was chosen by manually drawing the areas with CTIP2-positive cells in the scanned images of brain cortex using the Allen Brain Atlas (http://atlas.brain-map.org) as guidance. Areas with tissue creases or ruptures were excluded from the region of interest before the automatic cell detection step. From every mouse, one out of two sections from both brain hemispheres were randomly chosen for the blinded analysis. CTIP2-positive cells were counted and normalized to mm^2^ of cortical area. For the detection of DAPI-NeuN-positive cells (*n* = 2/genotype/sex), the StarDist heavy augment detection model was applied [23] within a QuPath Groovy script. The silhouettes of the cortex, medulla, and cerebellum were manually annotated based on the Allen Brain Atlas and exported to a JSON file. All DAPI-positive nuclei detections were exported as a separate JSON file for each image. In a second Groovy script, both the annotations and detections were imported into the corresponding FITC images from the JSON files. The DAPI-positive detections were further classified based on the mean green fluorescence intensity in the nucleus, using a threshold filter of 7, to calculate the number of DAPI-FITC-positive nuclei per mm² of section area. Double immunofluorescence co-localization studies were performed on brain sections using primary antibodies NeuN and LAMP1, LC3 and LAMP1, LC3 and SYP, IBA1 and LAMP1 as well as IBA1 and CD68 as listed in (Supplementary Table 8) after EDTA antigen retrieval (H2) in a BOND RX^m^ detection system. Sections were subsequently treated with secondary antibodies and nuclear counterstaining was achieved using 4′,6-diamidino-2-phenylindole. Sections were imaged with a NanoZoomer S60 scanner (Hamamatsu, Japan) and the expression of each protein labeled with corresponding fluorochrome was captured separately and then merged. Spinal cord was dissected from formalin fixed tissues. Cervical part of the spinal cord was then immersed in 30% sucrose solution for 48 h at 4 °C followed by embedding in freezing media (tissue-Tek OCT #4583) in a freezing block and stored at –80 °C till sectioning. Frozen spinal cord was subjected to cryo-sectioning at 20 µm thickness and the sections were mounted to slides. Sections mounted on slides were blocked for 1 h then stained with primary antibody overnight at 4 °C followed by secondary antibody staining, Hoechst staining and mounting in Prolong Gold supplemented with DAPI media. Pathological lesions were evaluated descriptively and their prevalence was compared between genotypes. The number of Ctip2- and NeuN-positive cells was compared using a two-sided Welch *t*test.

### Vibratome sectioning of brain

12-month-old ki/ki and wt/wt were perfused in a PFA/GA fixative (2% PFA, 2.5% glutaraldehyde, 2 µM calcium chloride, 0.1 M cacodylate buffer) at a flow rate of 1.3 ml/min. Post perfusion whole brain was removed and fixed in PFA/GA fixative for 12 h at 4 °C. Post fixation, brains were subjected to vibratome (Leica VT1200S) sectioning of 100 µm thickness. The sections were then stored in 0.1 M cacodylate buffer at 4°C till processed for EM.

### Brainstem scanning and electron microscopy

Sagittal vibratome sections were further dissected to a maximum size of 1 mm² containing the region of interest. These samples were stained following a standard reduced osmium, thiocarbohydrazide (TCH), osmium (rOTO) *en bloc* staining protocol [24]. In brief, post-fixation was achieved by incubation in 1% osmium tetroxide (EMS), 1% potassium ferrocyanide (Sigma) in 0.1 M sodium cacodylate (Science Services) buffer (pH 7.4) for 1 h, followed by one wash in buffer and two washes in distilled water, for 10 min each. Osmium tetroxide staining was enhanced by reaction with filtered 1% aqueous TCH (Sigma) for 45 min at 40 °C. After incubation in TCH, the samples were washed in water thrice and incubated in 1% aqueous osmium tetroxide for 1 h, washed in distilled water and further contrasted by overnight incubation in 1% aqueous uranyl acetate at 4 °C and 2 h at 50°C the next day. Samples were dehydrated in a graded series of ethanol followed by three 20 min incubations in dry acetone. Next, samples were infiltrated with increasing concentrations of LX112 (LADD) resin in acetone. These steps were: 25% and 50% resin for 2 h each, 75% resin overnight and two 100% resin steps for 2 h each. Subsequently, samples were polymerized at 60°C for 48 h.

For scanning electron microscopy, the samples were ultrathin sectioned at a thickness of 100 nm (UC7 Ultramicrotome; Leica) and collected onto carbon coated Kapton tape pieces. Sections were imaged using a Crossbeam340 (Zeiss) at 8 kV with an angular BSD detector.

For transmission electron microscopy, the samples were ultrathin sectioned at a thickness of 50 nm (UC7 Ultramicrotome; Leica) and collected TEM-Grids (Science Services). Sections were imaged using a JEM 1400 plus (Jeol) at 120 kV equipped with a TemCam-XF416 Camera (TVIPS) operated by the EMplified software (TVIPS).

### Transcriptome analysis

Total RNA was isolated from mice brain (*n* = 8/ genotype) employing the RNeasy Mini kit (Qiagen, Hilden, Germany) including Trizol treatment. The Agilent 2100 Bioanalyzer was used to assess RNA quality and RNA with RIN > 7 was used for transcriptome analysis. Total RNA was analyzed by RNA sequencing done in the core facility of the Helmholtz Zentrum München with the Illumina® Stranded Total RNA Prep, Ligation with Ribo-zero plus on an Illumina NovaSeq sequencer with a PE100 stranded protocol, resulting in an average of 24 million reads per sample. Paired-end data was analyzed by a RNAseq pipeline consisting of quality control (FastQC, MultiQC), read trimming (trim_galore v0.6.10, cutadapt v3.4), genome alignment (STAR v2.7.10b, genome GCRm39_109, Dobin et al., 2013), and gene-level read counting (summarizeOverlaps, mode = ’IntersectionNotEmpty’, package GenomicAlignments v1.28.0). Significantly regulated genes were determined in R (v4.3.0) with DESeq2 (v1.40.2, Love MI et al. 2014) after excluding low expressed genes (counts >16 across all samples). The p-values attained by the Wald test were corrected for multiple testing using the Benjamini and Hochberg method (padj). Gene sets were filtered for counts >5 in at least half of the samples in at least one group. For the biological interpretation of the significant gene regulation (raw *p* < 0.01), we performed pathway enrichment analyses through the use of QIAGEN’s Ingenuity Pathway Analysis software (IPA®, QIAGEN Redwood City, content v94302991). Selected terms with *p* < 0.05 were manually classified into categories.

### Gene expression profiling using nanostring

Sub-dissected snap-frozen brain materials from mice (12 months, *n* = 3 males/ genotype) were mechanically powdered in liquid nitrogen. Qiagen RNeasy Mini Kit, was used for total RNA isolation from Forebrain (cerebrum) and Hindbrain (cerebellum and medulla). 100 ng total RNA per sample was used for gene expression analysis with the NanoString Glial Profiling panel. NanoString nCounter reads for all samples were analyzed and normalized using the nSolver software, including the R plugin for advanced gene set analysis and ROSALIND platform. The mean of each group was used for calculation of the fold changes. Each group contained three animals as biological replicates. Volcano plots were generated by plotting the log_10_ *p* values between wild-type and TECPR2-mutant group, against the calculated Log_2_ fold change values between the groups.

### CSF collection and sample preparation

CSF was collected from anesthetized mice (12months, *n* = 5, 3 females/genotype, 2 males/genotype) using medetomidin (0.5 mg/kg), midazolam (5 mg/kg) and fentanyl (0.05 mg/kg) by puncturing the cisterna magna with a self-pulled cannula using a surgical stereotax. The CSF was subsequently centrifuged at 1000 × *g* for 5 min, visually checked for blood contamination and frozen in 5 µl aliquots at –80 °C. Sample preparation and mass spectrometric analysis were done following published protocols with minor modifications [25, 26]. 5 µl murine CSF per sample was mixed with 2.75 µl denaturation buffer 0.5% Sodium Deoxycholate (5 mg/ml, SDC) in 250 mM ammonium bi-carbonate (19.76 mg/mL; NH_4_HCO_3_, ABC) and proteins reduced by addition of 2 µl 10 mM dithiothreitol (DTT) in 50 mM (ABC) for 30 min at room temperature (RT). Following alkylation by addition of 2 µl 55 mM iodoacetamide (IAA) for 20 min at RT in the dark, the reaction was quenched with 2 µl of 10 mM DTT. Samples were digested by incubation with 0.1 µg LysC in 0.1% SDC and 50 mM ABC for 3 h at RT, followed by tryptic digestion with 0.1 µg Trypsin in 0.1% SDC and 50 mM ABC for 16 h at RT. Digestion was terminated by acidification with 150 µl 0.1% formic acid (FA) and 8 µl FA and SDC removed by centrifuged at 16,000 × *g* for 10 min at 4 °C. Supernatants were desalted by STAGE Tip purification as previously published [27]. In brief, samples were loaded on two pre-equilibrated and washed C18 Empore disks, washed 4 times with 0.1% FA and eluted with 40 µl of 80% acetronitrile (ACN) and 0.1% FA. Samples were dried using a speed vac and reconstituted right before injection with 20 µl 0.1% FA.

### CSF proteomics

5 µl of CSF-derived peptide mix was separated on a nanoElute HPLC system (Bruker, Germany) coupled to a TimsTOF pro mass spectrometer (Bruker, Germany) with a CaptiveSpray ion source (Bruker, Germany). Separation was performed on a self-packed C18 analytical column (15 cm × 75 µm ID, ReproSil-Pur 120 C18-AQ, 1.9 µm, Dr. Maisch GmbH) using a binary gradient of water (A) and ACN (B) containing 0.1% FA at a flow rate of 300 nl/min (0 min, 2% B; 2 min, 5% B; 62 min, 24% B; 72 min, 35% B; 78 min, 85% B, 82 min 85% B) and a column temperature of 50 °C. Mass spectra were acquired using data independent acquisition parallel accumulation-serial fragmentation (DIA-PASEF). Ion accumulation and separation using trapped ion mobility spectrometry (TIMS) was set to a ramp time of 100 ms, one scan cycle included one TIMS full MS scan and 26 windows with a width of 27 m/z covering a m/z range of 350–1002 m/z. Two windows were recorded per PASEF scan at a cycle time of 1.4 s in positive mode. CSF proteomes were analyzed using DIA-NN (version 1.8) [28] with an 0.01 FDR filtering using library-free search on an in-silico spectral library derived from a full mouse proteome. Number of missed cleavages was set to 2, peptide length to 7–30 and precursor charges to 1–4. Cysteine carbamidomethylation was set as a fixed modification, the number of variable modifications to 2 and included UniMod:35 and UniMod:1. Match-between-runs and retention time-dependent cross-run normalization was enabled.

### Medulla proteomics

Brain from perfused mice (17 months, *n* = 4, 2 females/genotype, 2 males/genotype) were extracted and fixed in 4% PFA followed by embedding in freezing media (tissue-Tek OCT) in a freezing block and stored at –80 °C till sectioning. Frozen brains were subjected to sagittal cryo-sectioning at 50 µM thickness. 2X circular stamps of 2 mm diameter each were cut out from the distal part of Medulla. Both the stamps were collected in a single tube and lysed in 30 µL of lysis buffer containing 0.2% n-Dodecyl-β-D-Maltoside (DDM) and 100 mM Triethylammonium bicarbonate (TEAB) pH 8.5. Lysates were heated at 95°C for 60 min followed by sonication with Covaris and spun down. Then supernatants were treated with sequentially with 12% ACN, followed by Tris 2-carboxyethyl phosphine (TCEP) 10 mM and Chloroacetamide (CAA) mix (5 mM/20 mM) and finally with 20 mM CAA. The treatment mix were heated again at 75 °C for 60 min. Digestion mix of 150 ng LysC and 300 ng Trypsin were added per sample and digested overnight at 37 °C. Digested samples were acidified using 8% FA and concentrated using speed vaccum. They were further acidified using 0.1% FA followed by desalting using C18 STAGE tips. Peptides were eluted two times from the tips using 80% ACN for 15 min each. Eluted peptides were concentrated using speed vac and dissolved in 13 µL 0.1% FA. Concentration of peptides were measured using Qubit and 350 ng peptides injected into nanoElute HPLC system (Bruker, Germany) coupled to a TimsTOF pro mass spectrometer (Bruker, Germany) with a CaptiveSpray ion source (Bruker, Germany). MS acquisition and analysis were carried out similarly as described earlier in CSF proteomics.

### Microglia isolation

Primary microglia cells were isolated from 12-month-old ki/ki and wt/wt mice. Whole brain was harvested and dissociated using an adult brain dissociation kit (Miltenyi Biotec 130-107-677) in a gentleMACS™ Octo Dissociator with Heaters (Miltenyi Biotec 130-096-427). Following a debris removal step, cell suspension from brain was incubated with microglia specific CD11b magnetic beads. The suspension was then passed through LS columns bound to a MACS separator. Only cells that were labeled with CD11b magnetic beads bound to the magnetic column when coupled to the separator. Beads coupled microglia were then eluted from the column by removing it from the separator using a PB buffer (0.5% BSA in DPBS). Cells were counted and plated on cover slips in recombinant gm-CSF (rndsystems 415-ML-020) supplemented DMEM/F12 media (Gibco 31330-038) and was cultured for a week to recover before performing immunofluorescence studies. Cells used for whole cell proteomics analysis were pelleted and stored at –80 °C till they were processed.

### Microglia proteomics

Microglia isolated from two ki/ki and two wt/wt mice were pooled as one biological replicate (12 months, *n* = 4 females/genotype). Cell pellets were lysed in Urea buffer 9 M Urea, 50 mM Tris pH 8, 150 mM NaCl, 1x protease inhibitors (Roche) at 4 ^o^C for 30 min. Cell lysate was sonicated for 60 s followed by centrifugation at 2500 × *g*. Protein concentration was measured and equalized using a BCA assay. Proteins were first reduced with 5 mM DTT (Sigma) at 55 ^o^C for 30 min, followed by alkylation with 14 mM IAA (Sigma) at room temperature (RT) for 30 min in dark and finally quenched with 5 mM DTT for 15 min. Samples were further diluted 1:5 with 25 mM Tris-HCl, pH 8.2 and were digested first with LysC (FUJIFILM, 2 µl/100 µg protein) at RT for 3 h followed by overnight tryptic digestion (0.5 µg/100 µg protein) at 37 ^o^C. Digested peptides were acidified with 1% trifluoracetic acid and concentrated using a vacuum centrifugation. Concentrated peptides were further acidified by the addition of 100% acetic acid to a pH<2.and then fractioned by C18-SCX custom made STAGE tips (2x SCX at bottom and 2x C18 on top) as described earlier (Rappsilber, J et al. 2007). Samples were eluted stepwise in increasing concentration of NH4AcO in elution buffers (20 mM to 500 mM NH_4_ACcO in 0.5% acetic acid and 20% ACN). Fractions were further desalted on custom made C18 STAGE tips and then eluted in 0.1% FA. Peptide mixtures were separated on a 140 min ACN gradient in 0.1% FA at a flow rate of 400 nl/min (3–6% ACN gradient for 2 min, 6–30% ACN gradient for 20 min, 44-75% ACN for 10 min, 75–100% ACN gradient for 5 min). Peptides were identified in full MS/ddMS^2^ (Top 15) mode, dynamic exclusion was allowed for 20 s and identifications with an unassigned charge or charges of one or <8 were rejected.

### Cell culture and transfections

HeLa and BV2 cells were grown in Dulbecco´s modified Eagle medium (DMEM Gibco, #6195-026) supplemented with 10% fetal bovine serum FBS9 and 1% sodium pyruvate. Stable cell lines were grown in medium supplemented with puromycin (2 µg/ml or 4 µg/ml Sigma, #P8833). Transient transfection of cells was performed using PEI (polyethylenimine, Polysciences Europe GmbH) or Lipofectamine 2000 (Invitrogen) according to manufacturer’s protocol for 24–48 h.

### Cloning and cell line generation

Gateway cloning was used for cloning of all genes. ORFs were amplified with respective primer pairs containing attB flanking sites and cloned into an entry vector pDONOR233. Donor clones were then transferred to expression vectors such as pHAGE HA-flag, pDEST-V1(Snapgene-73635), pDEST-V2(Snapgene-73636), pHAGE GFP using homologous recombination. pHAGE HA TECPR2 full-length (FL) and fragments were used for stable transfection in HeLa cells using Lentiviral transduction. Lentiviral particles were generated by transfection of 293 T cells in a ratio of 1 µg DNA to 3 µl transfection reagent (Lipofectamine 2000). Recipient HeLa cells were incubated with viral particles mixed with polybrene for 24 h followed by selection with 2 µg/ml Puromycin. Post selection cells were always cultured in DMEM media supplemented with Puromycin. For CRISPR mediated KO of TECPR2 in HeLa cells, guide RNAs were designed using the Broad Institute gRNA designer tool targeting exon 2 and 5 of TECPR2 gene. The two guide RNAs were cloned individually in pSpCas9(BB)-2A-Puro (PX459) vector using BbsI restriction enzyme. Vector containing each guide RNA was co-transfected in HeLa cells using transfection reagent (X-tremeGENE HP DNA, Roche#6366244001) for 48 h followed by selection with media containing puromycin for another 48 h. Post selection cells were cultured in DMEM media without any selection. KO was confirmed by immunoblotting. Cells were further subjected to single cell clonal selection by serial dilution. Single cell clones were propagated and further validated for knock-out using western blot and genomic DNA sequencing. For CRISPR mediated KO of TECPR2 in BV2 cells, commercially available guide RNA (Dharmacon) targeting Exon 4 and 10 cloned in lentiviral vectors were used to generate lentiviral particles and then stably transduced in BV2 cells followed by selection with 4 µg/ml Puromycin. KO was confirmed as above. For EndoIP cell lines, commercially available pHAGE-3Flag-RAB5A (Snapgene-176488) was used to generate lentiviral particles and then stably transduced in Hela parental and TECPR2 KO cells. Post transduction cells were selected and maintained in Puromycin containing media as described earlier.

### Antibodies

All primary antibodies used for immunoblotting (IB) are used in a dilution of 1:1000 diluted in I-Block (Thermo scientific) and 1:100–1:500 for immunofluorescence (IF) diluted in 0.1% BSA in PBS. Secondary antibodies were used at a dilution of 1:10,000 or IB, and 1:500 for IF. All antibodies are listed in the Supplementary Table 8.

### Immunoblotting

For analysis of total proteins, cells were lysed in MCL-buffer (50 mM Tris pH 7.4, 150 mM NaCl, 0.5% NP40, 1x protease inhibitors (Roche), 1x PhosStop(Roche)) for 30 min at 4 ^o^C. Cell lysate were cleared by centrifugation at 20,000 × *g* for 10 min. Protein concentration of cleared lysates was measured using BCA assay and equalized. Adjusted protein lysates were then boiled in sample loading buffer (200 mM Tris-HCl, 6% SDS, 20% Glycerol, 0.1 g/ml DTT and 0.01 mg Bromophenol Blue). Proteins were separated by SDS-PAGE and transferred to nitrocellulose membranes. Membranes were blocked in 0.1% Tween-20 supplemented iBLock at RT for 1 h. Membranes were further incubated in primary antibodies (as indicated in the list) diluted in blocking buffer over-night at 4^o^C. Then the membranes were washed with TBS-T buffer, followed by incubation in secondary-HRP conjugated antibodies for 1 h at RT, washed again with TBS-T and immediately subjected to enhanced chemiluminescence analysis.

### TECPR2 interaction mapping

For mapping interaction of TECPR2, stable cell lines expressing HA tagged FL and different variants were used. Cells were harvested by trypsinization and cell pellets were lysed in MCL-buffer (50 mM Tris pH 7.4, 150 mM NaCl, 0.5% NP40, 1x protease inhibitors (Roche), 1x PhosStop (Roche)) or Glycerol IP buffer (20 mM Tris pH 7.4, 150 mM NaCl, 5 mM EDTA, 0.5% TritonX,1x protease inhibitors (Roche), 1x PhosStop (Roche), 10 glycerol) at 4 ^o^C for 30 min. Cell lysate were cleared by centrifugation at 20,000 × *g* for 10 min. Protein concentration of cleared lysates was measured using BCA assay and equalized. 10% of adjusted protein lysate was retained as an input fraction. Pre-equilibrated HA agarose (Sigma) was added to rest of the lysates and samples were incubated overnight at 4 ^o^C with gentle rotation for immuno-precipitation (IP). Beads were then washed extensively with the same lysis buffer. Input fraction and IP fraction were boiled in sample loading buffer (200 mM Tris-HCl, 6% SDS, 20% Glycerol, 0.1 g/ml DTT and 0.01 mg Bromophenol Blue) followed by immunoblotting.

### Bimolecular complementation affinity purification (BiCAP)

pDEST-V2-VPS41 only or pDEST- V1-TECPR2 FL and pDEST-V2-VPS41 was co-transfected in Hela TECPR2 KO cells using Lipofectamine 2000 for 24 h. Cells were harvested by trypsinization and cell pellet was lysed in GFP IP buffer (50 mM Tris-HCl, pH 7.5, 150 mM NaCl. 0.5 mM EDTA, 0.5% NP40, 1x protease inhibitors (Roche), 1x PhosStop (Roche)) at 4 ^o^C for 30 min. Cell lysate was cleared by centrifugation at 20,000 × *g* for 10 min. Protein concentration of cleared lysates was measured using BCA assay and adjusted. Pre-equilibrated GFP-Trap-A (Chromotek) beads were incubated with protein lysates overnight at 4 ^o^C with gentle rotation for pull down. Beads were extensively washed with lysis buffer devoid of detergent. On beads tryptic digestion was carried out as per manufacturer protocol. Protein bound to beads was incubated in elution buffer I (50 mM Tris pH 7.5, 2 M Urea, 1 mM DTT, 0.02 µg/µl Trypsin) at 30 ^o^C at 400 rpm for 30 min, centrifuged at 2500 × *g* for 2 min at 4 ^o^C. Supernatant was transferred to a new tube. Beads were again incubated twice with elution buffer II (50 mM Tris-Hcl pH 7.5, 2 M urea, 5 mM iodoacetamide) and eluate were combined with previously collected supernatant. Trypsin digestion was allowed to continue overnight at 30 ^o^C at 400 rpm. Reaction was stopped by addition of 1 µl trifluoracetic acid. Digested peptides were dried by vacuum centrifugation, resuspended in 30 µl 5% acetonitrile (Roth)/1% TFA (Fluka), desalted on custom made C18 STAGE tips and reconstituted in 0.1% FA.

### EndoIP

Hela parental and TECPR2 KO cells stably overexpressing pHAGE-3Flag-RAB5A was used. Cells were washed twice with PBS, harvested and suspended in cold endo-IP buffer (50 mM KCl, 100 mM KH_2_PO_4_ and 100 mM K_2_HPO_4_ pH7.2, supplemented with protease inhibitors). Cell suspension was homogenised with a tight-fitting pestle and centrifuged at 1500 × *g* for 10 min, supernatants were then incubated with pre-equilibrated anti-FlagM2-affinity gel beads (Sigma #A2220) and rotated for 1 h at 4 ^o^C. Beads were then washed thrice with salt buffer (Endo IP buffer supplemented with 300 mM NaCl) and endosomal proteins were eluted by incubating beads in 150 µl Urea buffer (8 m Urea, 50 mM tris pH8, 150 mM NaCl) for 30 min shaking at 4 ^o^C and sonicated for 5 min. Eluted proteins were reduced with 5 mM TCEP (Sigma) at 55 ^o^C for 30 min, alkylated with 15 mm IAA(Sigma) at Rt for 30 min and finally quenched with 10 mM DTT (Sigma) at RT for 15 min. Proteins were then precipitated using methanol chloroform precipitation. Precipitated proteins were reconstituted in 30 µl 50 mM ABC buffer and subjected to overnight tryptic (1 µg per sample) digestion. Digestion was stopped by addition of 30 µl 50% ACN (Roth) / 5% FA (Merck). Digested peptides were dried by vacuum centrifugation, resuspended in 30 µl 5% ACN (Roth) / 1% TFA (Fluka), desalted on custom made C18 STAGE tips and reconstituted in 0.1% FA Peptides reconstituted in 0.1% FA are separated using an Easy-nLC 1200 liquid chromatograph (Thermo Scientific) followed by peptode detection on a Q exactive HF mass spectrometer (Thermo Scientific). A custom-made fused silica capillary packed with C18AQ resin (Reprosil-PUR 120, 1.9 µm, Dr. Maisch) was used for separation of samples. Peptide mixtures were separated on a 35 min acetonitrile gradient in 0.1% FA at a flow rate of 400 nl/min (5–38% ACN gradient for 23 min, 38–60% CAN gradient for 3 min, 60–95% ACN gradient for 2 min).

### Processing of MS data

Raw data were analyzed using MaxQuant (version 1.6.0.1) [29] in reversed decoy mode based on a human or murine reference proteome (Uniprot-FASTA, UP000005640 or UP000000589) with false discovery rate of 0.01. During analysis, label-free quantification, re-quantification and match- between-runs were enabled and atleast 4 biological replicated were analyzed. Max Quant Protein group files were further processed using Perseus (version 1.6.5.0) [30]. Generally, common contaminants, reverse and site-specific identifications as well as proteins identified with a peptide count <1 or <2 were excluded. Rest of the protein groups were filtered by statistical testing as mentioned. DAVID (version 6.8) was used for functional enrichment analysis.

### Immunofluorescence

Primary microglia and BV2 cells were grown on poly-lysine (Sigma) coated coverslips, HeLa cells on uncoated coverslips. Cells were washed with DPBS (Gibco), fixed with 4% paraformaldehyde (Santa Cruz) for 10 min and then permeabilized with either 0.5% Triton X-100 (Merck) or 0.1% Saponin plus 0.1% Triton X for 10 min. Cells were then blocked with 1% BSA for 1 h at RT followed by sequential incubation of primary and Alexa coupled secondary antibodies for 1 h each at RT in dark. Coverslips were mounted in Prolong Gold supplemented with DAPI (Invitrogen). For Dextran assay, microglia were incubated with 150 µg/ml Dextran Alexa 568 along with 1 µM SiR (Spirochrome # SC012) for 3 in culture, washed 4 times with DPBS, then fixed and mounted with in Prolong Gold supplemented with DAPI (Invitrogen). For primary microglia staining, cover-slips were additionally treated with True Black to block Lipofuscin signal before mounting. Imaging was done on a LSM800 confocal microscope (Zeiss) with a 63x oil-immersion objective. Images were first processed with Zen blue (Zeiss v 2.5) followed by quantification using ImageJ.

### EGF assay

Equal number Hela parental and TECPR2 KO cells were plated on cover slips in complete DMEM media. After 24 h, media was changed to serum deprived DMEM media for 2 h following addition of 100 ng/ml EGF-Rh (Thermofisher, #E3481) and 500 nM SiR for 1 h. Next, cells were washed 2 times with PBS and incubated for 3 h in serum deprived DMEM media. Cells were fixed in PFA as described earlier and imaged for EGF-Rh and SiR. Images were loaded in ImageJ and subjected to background subtraction, Log3D filter application, thresholding, region of interest (ROI) selection, and measuring particles per cell. Particles were measured in at least 30 cells over 3 biological replicates. For co-localisation analysis JaCop plugin was used, after thresholding of images Mander’s coefficient were calculated on ROI for 30 cells in 3 biological replicates. For quantification, 2-sides T-test was performed in Graph pad Prism 10 software and the values were plotted in graphs. Images were first processed with Zen blue (Zeiss v 2.5) followed by quantification using ImageJ.

### Image analysis

For counting total particles per cell, images were loaded in ImageJ and subjected to background subtraction, Log3D filter application, thresholding, region of interest (ROI) selection and measuring particles per cell. Particles were measured in at least 30–60 cells over 3 biological replicates. To analyze relative signal intensity of cells, images were loaded in ImageJ and region of interest (ROI) selected. Selected ROI were subjected to measure intensity function in ImageJ followed by subtraction of average background intensity. Relative intensity ratios were generated by dividing individual ROI intensities by average intensity of control cells. Intensities were measured in at least 30–60 cells over 3 biological replicates. To calculate percentage of cells with peri-nuclear endo-lysosomal marker clustering, cells from at least 5 different fields per condition per replicate were counted using cell counter function in ImageJ and marked as either clustered or un-clustered cell. Percentage of clustered cells per field were then calculated as a ratio of clustered cells per field to the total number of cells per field multiplied by 100. Total 40–60 cells were counted over 3 biological replicates. For co-localization analysis JaCop plugin was used, after thresholding of images Mander’s coefficient were calculated on ROI for 30–60 cells in 3 biological replicates. For statistics, 2-tailed paired *t* test was performed between conditions in Graph pad Prism 10.5.0 software, bar graph with standard error of mean with or without individual data points were plotted and p-values were denoted in the plots.

## Results

### HSAN9 mutation knock-in mice show loss of TECPR2 and altered behavior

**Fig. 1:**
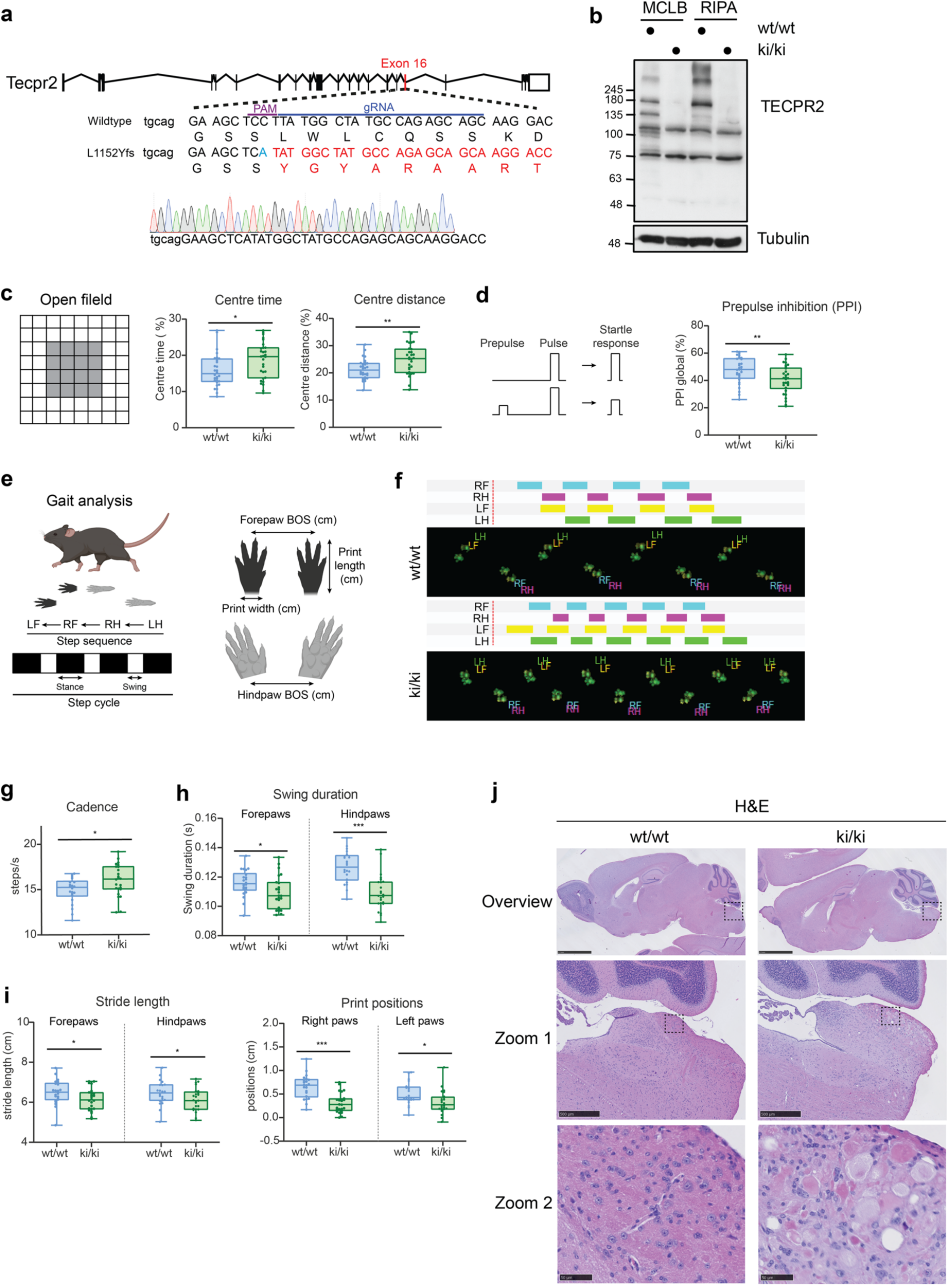
Behavioral and histological characterization of *Tecpr2* ki/ki mice. **a** Schematics and genomic sequence depicting CRISPR/Cas9 target region in Exon 16 of *Tecpr2*. **b** Western blot validation of whole brain lysates prepared from 5-month-old *Tecpr2* ki/ki and wt/wt mice. **c** Time and distance traveled in open field central aversive zone during 20-min open field test with 5-month-old *Tecpr2* ki/ki and wt/wt mice (*n* = 30 per genotype, males *n* = 15, females *n* = 15, pooled). **d** % prepulse inhibition (PPI) indicating sensorimotor gating in 5-month-old *Tecpr2* ki/ki and wt/wt mice (*n* = 30 per genotype, males *n* = 15, females *n* = 15, pooled). **e** Catwalk XT® parameter descriptions. **f** Paw print patterns of a representative 5-month-old *Tecpr2* ki/ki and wt/wt male mice. **g** Catwalk run characteristics of cadence (steps/s) in 5-month-old *Tecpr2* ki/ki and wt/wt mice (10 male wt/wt, 12 female wt/wt, 12 male ki/ki, 12 female ki/ki, pooled). **h** Temporal Catwalk parameter of swing duration from mice analyzed in (**e**). **i** Spatial Catwalk parameters of stride length and print position from mice analyzed in (**e**). *p < 0.05, **p < 0.01, ***p < 0.001 genotype effect, 2-way ANOVA. **j** Representative brain sections stained with hematoxylin and eosin from 5-month-old *Tecpr2* ki/ki and wt/wt mice (*n* = 5/genotype/sex). Boxes show affected brain stem region. Scale bar: 2.5 mm (overview), 500 µm (zoom 1), 50 µm (zoom 2).

To generate a HSAN9 mouse model mimicking a nonsense mutation in patients, we employed CRISPR/Cas9 gene editing to introduce the TECPR2 frameshift mutation L1152Yfs in mice corresponding to L1139Rfs in humans (Fig. 1a). Gene editing was confirmed by genomic sequencing and homozygous knock-in mice (hereafter referred to as ki/ki mice) were fertile and born at Mendelian ratios. Immunoblot analysis of brain extracts from *Tecpr2* ki/ki mice and their wild-type littermates (wt/wt) revealed that Tecpr2 carrying the L1152Yfs mutation was not expressed at the protein level (Fig. 1b). Given the clinical manifestation of HSAN9 patients, 5-month-old *Tecpr2* ki/ki mice were subjected to a series of phenotypic assays including behavioral to characterize aspects of emotionality, sensorimotor function and coordination. In response to a novel, mildly stressful environment, *Tecpr2* ki/ki mice spent more time moving in the central aversive zone of the open field, indicative of decreased anxiety-related behavior (Fig. 1c). Sensorimotor gating ability was also impaired in *Tecpr2* ki/ki mice as indexed by decreased % prepulse inhibition (PPI) (Fig. 1d). Automated gait analysis with the Catwalk XT® system (Fig. 1e) unveiled that *Tecpr2* ki/ki mice exhibited clearly altered gait (Fig. 1f-i, Supplementary Fig. 1a–c). In particular, *Tecpr2* ki/ki mice featured quantitative increased walking cadence (Fig. 1g), decreased temporal and spatial walking parameters including swing duration, stride length and paw print position (Fig. 1h, i, Supplementary Fig. 1a, b) as well as a decreased footprint area of the forelimb (Supplementary Fig. 1c). A similar pattern of increased step number related to movement scaling occurs in Parkinson’s disease (PD) patients and is considered a compensatory response to postural instability [31]. Notably, all *Tecpr2* ki/ki mice showed no differences in the run characteristics of speed and duration (Supplementary Fig. 1d) and could adequately traverse the walkway without overt signs of muscle weakness, behavioral disturbance or body weight changes that would otherwise confound analysis of walking characteristics. Together, *Tecpr2* ki/ki mice phenocopy *Tecpr2* KO mice [13] with regard to the lack of TECPR2 expression and similar behavioral phenotypes, indicating that loss of TECPR2 function is likely a contributing disease mechanism.

### Loss of TECPR2 leads to neuroaxonal dystrophy in the medulla oblongata

Using hematoxylin and eosin (H&E) stained brain sections, we assessed the brain morphology of 5-month-old *Tecpr2* ki/ki mice. With complete penetrance, we observed alterations exclusively in the medulla oblongata of both sexes (*n* = 5 per genotype/sex), consisting of swollen hypereosinophilic axons (axonal ‘spheroids’) (Fig. 1j). These morphological lesions were not present in the medulla of aged-matched wildtype littermates and nowhere else in *Tecpr2* ki/ki mouse brains. The spheroids varied in size and shape, with some displaying granular positivity for Periodic Acid-Schiff (PAS) staining (Supplementary Fig. 1e), suggesting the presence of accumulated glycoproteins or proteoglycans. Notably, spheroids in sensory regions of the brain are typical in neurodegeneration [32] and have also been observed in *Tecpr2* KO mice [13]. Importantly, spheroids were detectable but less frequent in the medulla of 2-month-old *Tecpr2* ki/ki mice (Supplementary Fig. 2a), indicating that spheroids form progressively. Consistent with autosomal-recessive or compound heterozygotic inheritance of HSAN9, spheroids were absent from the medulla of 2-month-old heterozygote *Tecpr2* wt/ki mice (Supplementary Fig. 2b). Hence, *Tecpr2* ki/ki mice show a similar age-dependent neuroaxonal dystrophy in the medulla oblongata as reported for *Tecpr2* KO mice [13].

### Loss of TECPR2 leads to neurodegeneration, gliosis, and autophagy-lysosome defects

**Fig. 2:**
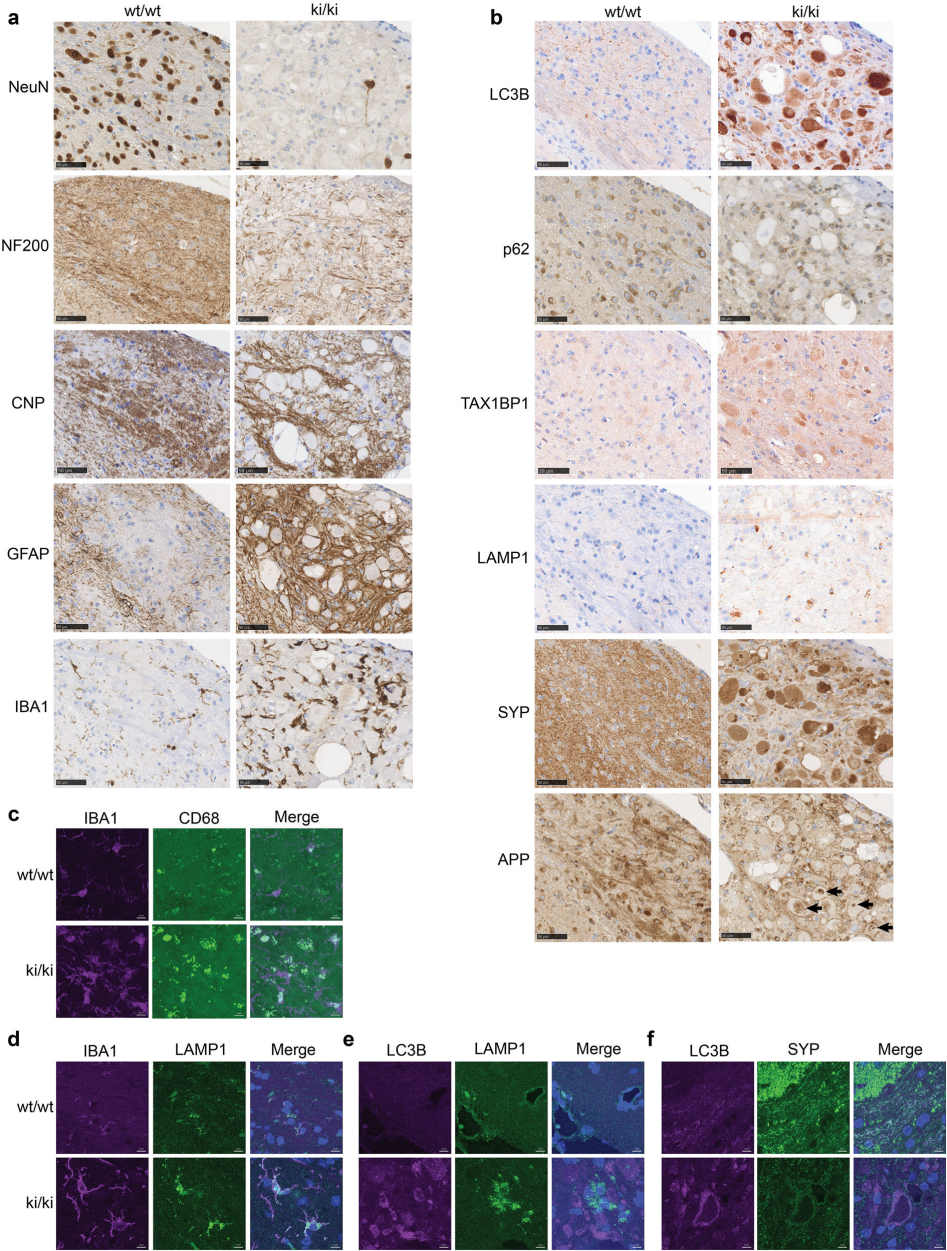
Pathological changes in the spheroid-containing medulla of *Tecpr2* ki/ki mice. **a** Immunohistochemical staining with indicated neuronal, glial, and oligodendrocyte markers of the brainstem area containing axonal swellings in 5-month-old *Tecpr2* ki/ki mice. Age-matched wt/wt littermates were used as control. Scale bar: 50 µm. **b** Immunohistochemical staining of the same area as in (a) with autophagosomal (LC3B, TAX1BP1, p62), endolysosomal (LAMP1, APP) and synaptic vesicle (SYP) markers (n = 5/genotype/sex). Scale bar: 50 µm. Colocalization analysis of indicated markers in brain stem sections of 12-month- (**c**) and 5-month-old (**d**–**f**) *Tecpr2* ki/ki and wt/wt mice (*n* = 1). Scale bar: 10 µm.

Consistent with the manifestation of neuroaxonal dystrophy, immunohistochemical analysis of NeuN (neuronal nuclear antigen), NF200 (large myelinated axons marker) and CNPase (oligodendrocyte marker) revealed a dramatic reduction in neuronal cell bodies, extensive decrease and fragmentation of neurofilaments and a decrease in oligodendrocytes in the medulla of all 5-month-old *Tecpr2* ki/ki mice (Fig. 2a, Supplementary Fig. 2c). Furthermore, this region also showed increased numbers of astrocytes (detected by GFAP) and enlarged microglial cells (immunostained by IBA1) with ramified processes (Fig. 2a), indicative of astrogliosis and microgliosis. In line with progressive formation of spheroids (Supplementary Fig. 2a), gradual reduced NeuN signal and increased GFAP and IBA1 levels were already detectable in the medulla of 2-month-old *Tecpr2* ki/ki mice (Supplementary Fig. 3a). This indicates that neuronal malfunction and gliosis likely progress hand-in-hand. Moreover, we found that the observed spheroids in the medulla stained positive for the autophagy marker MAP1LC3B (LC3B), the selective autophagy receptor TAX1BP1 (but not p62/SQSTM1), the presynaptic neurosecretory vesicle integral membrane glycoprotein synaptophysin (SYP) and to some extent also the C-terminal part of the amyloid-beta precursor protein (APP) (Fig. 2b), suggesting that autophagosomal-endolysosomal pathways might be compromised. Intriguingly, levels of the lysosomal membrane protein LAMP1 also increased in the medulla of *Tecpr2* ki/ki mice. However, this signal seems not enriched in spheroids (Fig. 2b). Confocal microscopy of medulla sections revealed that microglia were activated as they were positive for CD68 and contained an enlarged LAMP1-positive lysosomal compartment (Fig. 2c, d). The latter phenotype likely contributed to the observed increased expression of LAMP1 since it did not colocalize with LC3B detected in spheroids (Fig. 2e) or with neuronal cell bodies stained with NeuN (Supplementary Fig. 3b). Conversely, LC3B and SYP were indeed both present and partially colocalized in aberrant processes (Fig. 2f). Strikingly, the loss of neuronal cell bodies was neuroanatomically restricted to a defined area in the medulla, namely to the dorsal column nuclei such as the cuneate nucleus and the gracile nucleus (Supplementary Fig. 4a–d), since quantification of NeuN across the whole medulla in comparison to the cortex or the cerebellum did not reveal significant altered NeuN levels (Supplementary Fig. 4e). Fragmented neurofilaments and accumulated LC3 were similarly found restricted to the same sensory-related medulla area. Other brain regions such as the cortex, only exhibited milder yet still detectable alterations in GFAP and IBA1 (Supplementary Fig. 4a–d). Thus, loss of TECPR2 leads to highly region-specific alterations of neurons—possibly including autophagosomal-endolysosomal defects - to which astrocytes and microglia respond. Notably, the observed loss of cell bodies and axonal swelling might be manifested in different neuron populations.

### Loss of TECPR2 also affects brain regions connected to the medulla

**Fig. 3:**
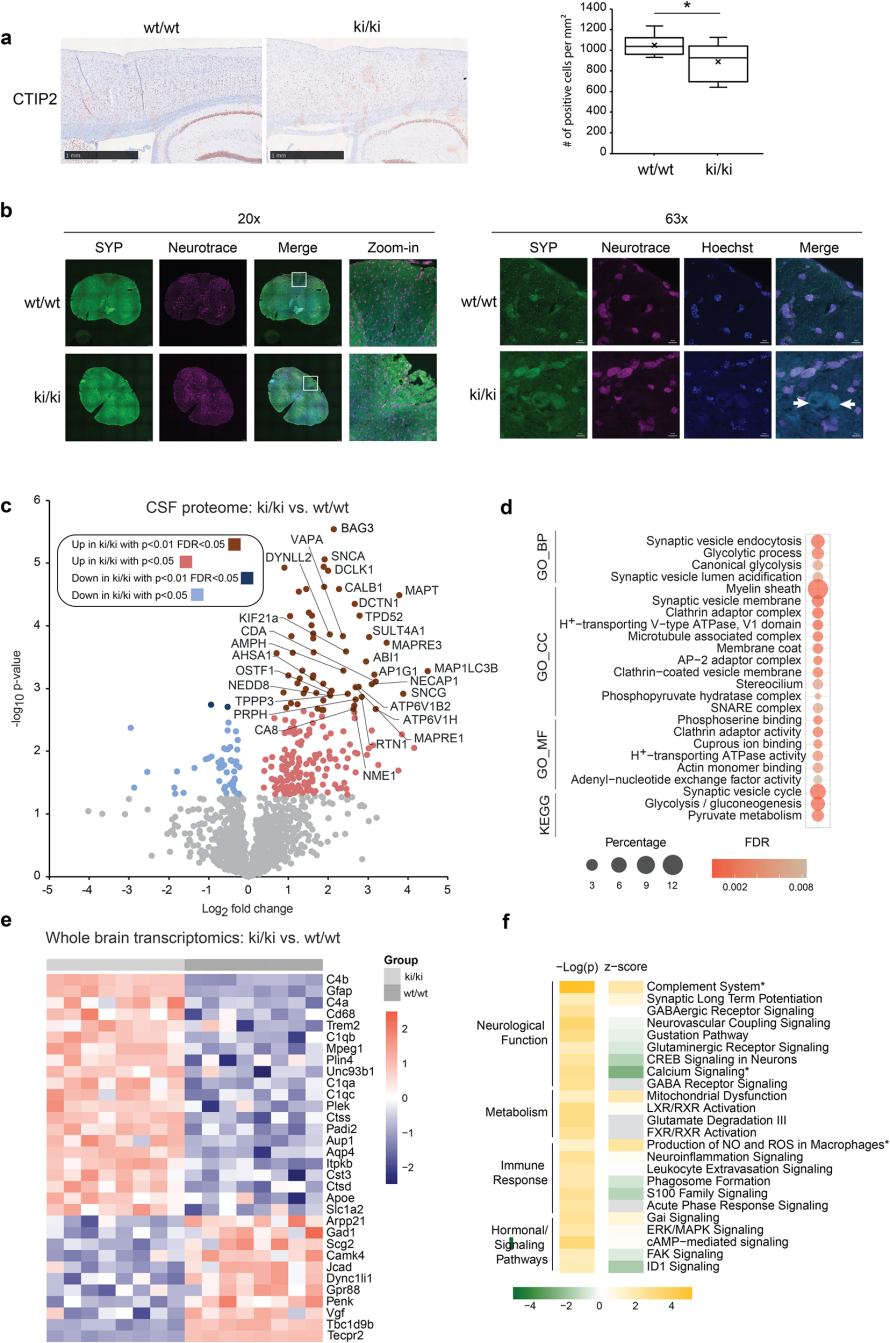
Changes in connected regions and at the global level in Tecpr2 ki/ki brains. **a** Quantitative immunohistology with CTIP2 in the cortex of 5-month-old *Tecpr2* ki/ki and wt/wt mice. (*n* = 10 per genotype, male *n* = 5, females *n* = 5, pooled) Scale bar: 1 mm. **b** Colocalization analysis of SYP and Neurotrace in the spinal cord of 5-month-old *Tecpr2* ki/ki and wt/wt mice (images acquired at 20 and 63x) (*n* = 2). Scale bar: 100 µm,10 µm. **c** Volcano plot of proteins with increased (red) or decreased (blue) abundance in cerebral spinal fluid (CSF) of 12-month-old *Tecpr2* ki/ki mice (*n* = 5, 3 females, 2 males). Proteins involved endolysosomal, autophagosomal and neurodegenerative processes are labeled. **d** GO term pathways enriched in CSF proteomics shown in (b). **e** Heatmap depicting up and down regulated genes detected by transcriptomics in whole brain tissue of 5-month-old *Tecpr2* ki/ki and wt/wt mice (*n* = 8x, males, padj <0.1). Upregulated and downregulated genes are marked in red and blue, respectively. **f** Selected significantly (*p* < 0.05) enriched canonical pathways based on regulated genes with *p* < 0.01. Z-scores >2 or <-2 indicate significant activation or inhibition of the respective pathway and are marked by “*”. Gray color was used if no z-score was available. Canonical pathways were manually classified into categories. Enrichment *p* values are shown as -Log_10_(*p* value).

Given the phenotype restriction to parts of the sensory-related medulla, we asked whether connected brain regions are also affected. Firstly, we turned to neurons originating in layer 5 of the cortex since these neurons are thought to project through the affected medulla area. Using CTIP2 as a specific marker for this long-distance neuronal cell population, we assessed neuronal loss in this particular cortex area. As expected, we found that projecting Ctip2-positive neurons were indeed significantly decreased (p < 0.05) (Fig. 3a). The fact that we also detected mild microgliosis in this area (Supplementary Fig. 4b) indicates that microglia might have already started to clear degenerating neurons projecting to the medulla. Secondly, we inspected the dorsal column of spinal cord sections subjacent to the affected medulla region. Staining for SYP - as a surrogate for the neuronal alterations observed in the medulla - revealed enlarged SYP-positive structures (Fig. 3b). Hence, the pathological changes are not strictly restricted to a defined part of the sensory-related medulla but extend to brain regions connected to this area.

### Loss of TECPR2 leads to altered CSF composition and transcriptome

Next, we performed proteomic analysis of cerebrospinal fluid (CSF) to monitor abundance changes in secreted or released proteins in response to the observed neuronal loss and axonal swelling. Across the five replicate *Tecpr2* ki/ki and wt/wt samples, we robustly detected 1788 proteins of which 206 and 46 were up and down regulated, respectively. Out of these 65 were significantly altered with 63 proteins increased and only two proteins decreased in abundance in *Tecpr2* ki/ki CSF (Fig. 3c, Supplementary Table 1). Strikingly, the three most strongly altered proteins LC3B, MAPT (also known as Tau) and γ-synuclein (SNCG) showed an 8- to16-fold increase in their protein levels. Gene ontology (GO) term enrichment analysis unveiled that proteins with increase abundance in the CSF were related to synapses, endocytosis, clathrin adaptors, V-type ATPase, cytoskeleton and myelin (Fig. 3d). Given that these terms correlate well with phenotypic changes observed by IHC (Fig. 2), it seems plausible that neurons whose cell body are lost or axons are swollen contribute to the altered CSF composition.

To unbiasedly detect changes in gene expression, we performed RNAseq-based transcriptomics of whole brain tissue from 5-month-old *Tecpr2* ki/ki and wt/wt mice. Stringent filtering and statistical analysis revealed 259 differentially regulated genes (p-values < 0.01) out of which 32 had an adjusted *p* values < 0.1 (Fig. 3e, Supplementary Table 2). While mRNA expression of Tecpr2 was robustly reduced as expected from the absence of Tecpr2 at the protein level (Fig. 1b), numerous microglia and astrocytes genes associated with neurodegenerative disease transcriptomic signatures [33] were significantly upregulated. Among them were the disease-associated microglia (DAM) genes Cd68, Trem2, Ctsd and ApoE, the astrocyte marker Gfap as well as multiple components of the complement system (i.e. C4a, C4b, C1qa, C1qb, C1qc) (Fig. 3e). Consistently, pathway analysis of the whole set of differentially regulated genes revealed an enrichment of the complement system, neuroinflammation signaling and phagosome formation among a number of several other neurologically relevant pathways such as Glutaminergic receptor, cAMP-responsive element-binding protein (CREB), Ca^2+^ and focal adhesion kinase (FAK) signaling as well as mitochondrial dysfunction (Fig. 3f).

### Defective organelles accumulate in the medulla of *TECPR2* knock-in mice

**Fig. 4:**
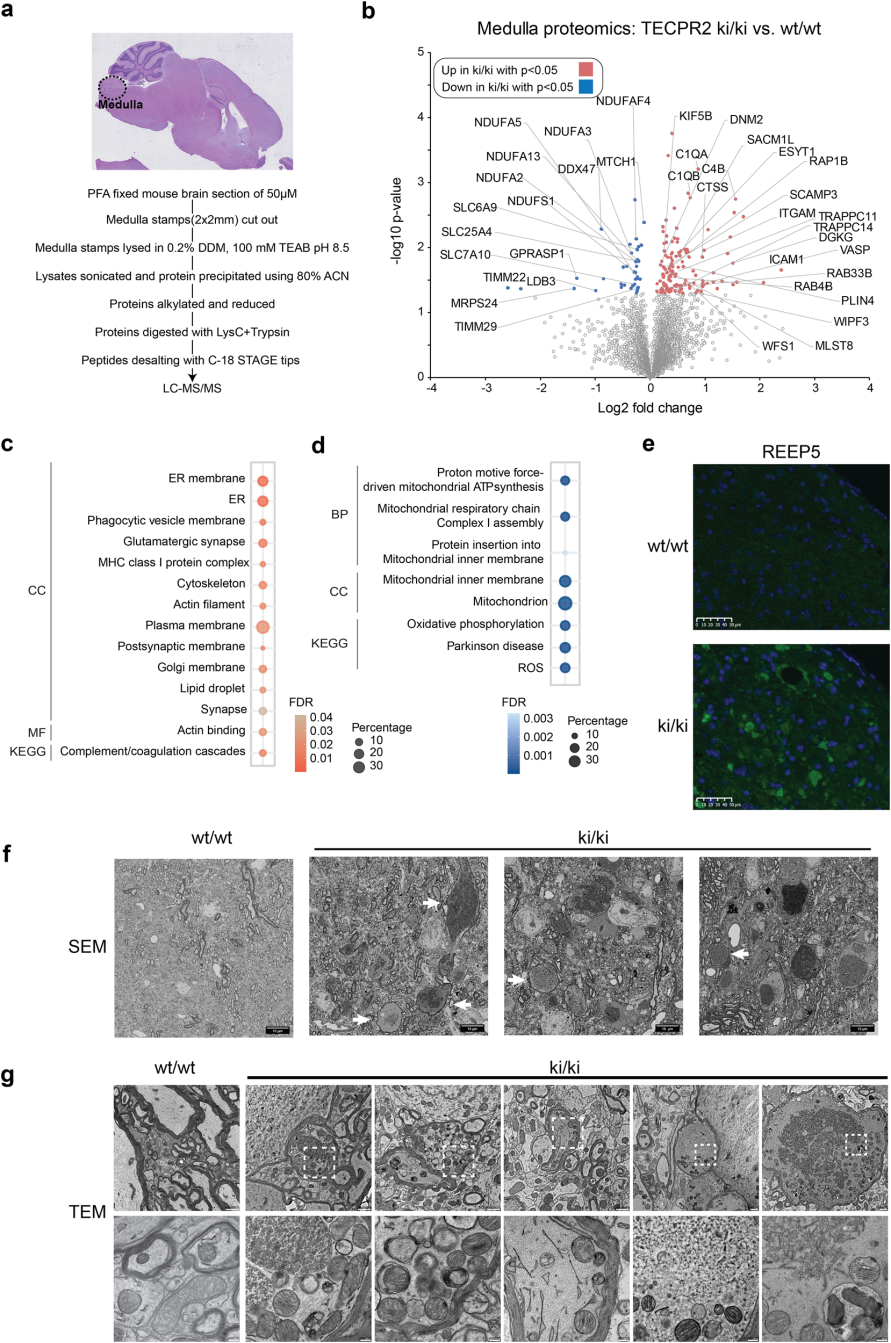
Proteomics and ultrastructural alterations in the medulla of T*ecpr2* ki/ki mice. **a** Medulla proteomics workflow. **b** Volcano plot of proteins whose abundance increase (red) or decrease (blue) in the medulla of 17-month-old *Tecpr2* ki/ki mice (*n* = 4, 2 females, 2 males). Proteins involved in endolysosomal, autophagosomal, mitochondrial and neurodegenerative processes are labeled. **c** GO terms enriched in the medulla proteome of *Tecpr2* ki/ki mice. **d** GO terms diminished in the medulla proteome of *Tecpr2* ki/ki mice. **e** Immunohistochemical stainings of spheroid-containing medulla with the ER marker REEP5. **f** Scanning electron microscopy (SEM) overview images of brain stem area from 5-month-old *Tecpr2* ki/ki and wt/wt mice. Scale bar: 10 µ). White arrows indicate swollen and aberrantly myelinated axons. **g** Transmission electron microscopy (TEM) images from samples shown in (**f**) depicting higher resolutions of different subcellular structures. Scale bar: 1 µm, 200 nm. White squares indicate area of higher magnification.

Complementarily, we also performed proteomics of the medulla. For this purpose, a defined area of the medulla was punched from brain sections of *Tecpr2* wt/wt and ki/ki mice and subjected to lysis. Proteins were precipitated and digested with trypsin followed by their analysis through liquid chromatography coupled to mass spectrometry (LC-MS) (Fig. 4a). Consistent with the transcriptomics, we observed an increase in complement components (C1qa, C1qb, C4b) and lysosomal enzymes (e.g. Ctss) as well as a decrease in mitochondrial proteins including subunits of the import machinery (Timm29, Timm22, Mtch1) and respiratory chain complex I (Ndufa2, Ndufa3, Ndufa5, Ndufa13, Ndufs1, Ndufaf4) (Fig. 4b, Supplementary Table 3). Corresponding GO terms including complement and coagulation cascade, oxidative phosphorylation, inner mitochondrial membrane and ATP synthesis featured prominently among regulated proteins (Fig. 4c, d). Besides, GO analysis also revealed an enrichment of secretory pathway-associated terms such as ER, Golgi, and plasma membrane (Fig. 4c, d) which have recently been shown to be altered in cells lacking TECPR2 [11]. Intriguingly, immunofluorescent microscopy of medulla sections from 2- and 5-months old *TECPR2* ki/ki not only confirmed the increase in abundance of the ER marker REEP5 but also showed that REEP5 colocalizes to spheroids (Fig. 4e, Supplementary Fig. 5a).

To corroborate our proteomics data, we performed electron microscopy. The spheroid-containing medulla of 12-month-old *Tecpr2* ki/ki mice contained several enlarged processes, most of which were myelinated axons (Fig. 4f). However, the myelin sheaths appeared considerably attenuated which is a sign of pathological axons. Strikingly, neuronal processes were filled with degenerated organelles in particular swollen mitochondria and enlarged ER but also lysosomes, single membrane vesicles and autophagosomes which accumulated in considerable numbers (Fig. 4g, Supplementary Fig. 5b). In addition, neuronal processes also contained excessive amounts of glycogen granules and amorphous aggregates (Fig. 4g). Collectively, the accumulation of these structures points to a defect in the autophagosomal-endolysosomal system in which either delivery to or degradation in lysosomes is compromised in medulla neurons. However, the fact that a specific subset of mitochondrial proteins decreased in abundance while mitochondria accumulate in swollen axons suggests that loss of TECPR2 might impact mitochondria beyond a potential function in mitophagy.

### Molecular signature of gliosis in *Tecpr2* knock-in mouse brains

**Fig. 5:**
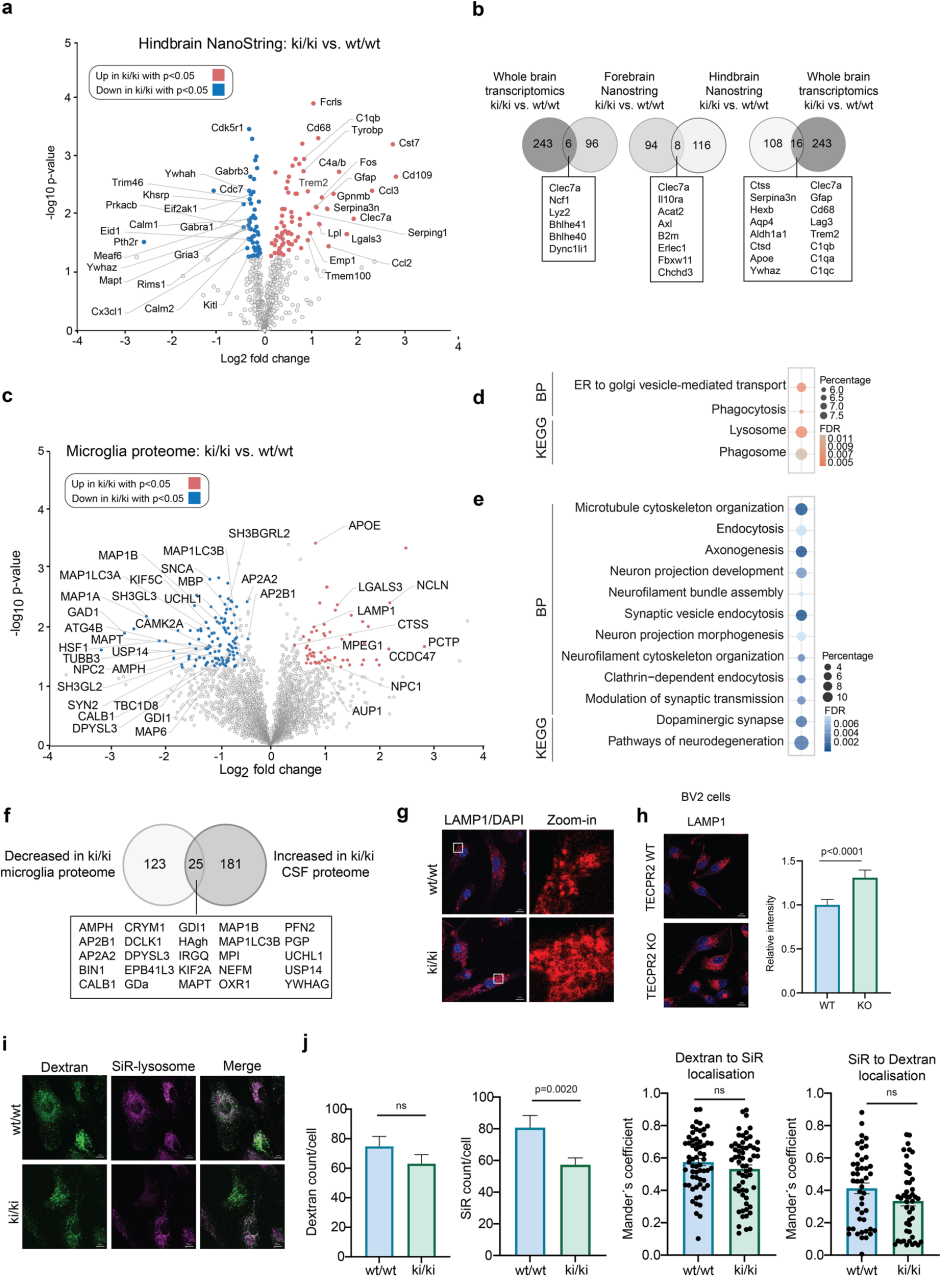
Analysis of *Tecpr2* ki/ki microglia. **a** Volcano plot of up- or downregulated genes detected by NanoString analysis in the hindbrain of 12-month-old *Tecpr2* ki/ki and wt/wt mice (*n* = 3, males). Genes with -Log_10_(p-value) ≥1.3 are highlighted in red (up) and blue (down). Top20 up- and downregulated genes are indicated. **b** Venn diagram depicting common genes significantly changed in whole brain transcriptomics, forebrain and hindbrain NanoString analyses. **c** Volcano plot of proteins up- (red) and downregulated (blue) in primary microglia derived from 12-month-old *Tecpr2* ki/ki and wt/wt mice (*n* = 4, females). Proteins involved in endolysosomal, autophagosomal and neurodegenerative processes are labeled. **d** GO terms enriched in Tecpr2 ki/ki microglia. **e** GO terms diminished in *Tecpr2* ki/ki microglia. **f** Venn diagram depicting common proteins whose abundance decreases in *Tecpr2* ki/ki microglia and increases in the CSF of *Tecpr2* ki/ki mice. **g** Microglia from *Tecpr2* wt/wt and ki/ki mice were isolated, cultured for 5 days, fixed and immunostained with an anti-LAMP1 antibody. DAPI was used to stain the nucleus. Scale bar, 10 µm. **h** *Tecpr2* WT and KO BV2 cells were fixed and immunostained with an anti-LAMP1 antibody. LAMP1 intensities were quantified (*n* = 3). **i** Microglia form *Tecpr2* wt/wt and ki/ki mice were isolated, cultured for 5 days, incubated with Dextran Alexa fluor 568 and SiR-lysosome for 3 h followed by fixation and confocal microscopy. Scale bar, 10 µm. **j** Quantification of Dextran and Sir counts from (**i**) (cells counted = 60, *n* = 3). Bar graphs show mean values with SEM as error bars.

Besides assessing neuronal consequences of TECPR2 loss we followed up on the gliosis phenotype (Figs. 2a and 3e). Thereto, we employed NanoString gene expression profiling to investigate inflammation-relevant gene expression patterns in the hind-and forebrain regions of *Tecpr2* ki/ki mice. While the former contains the spheroid loaded medulla, the latter encompasses the cortex with decreased numbers of Ctip2- positive neurons. Interestingly, TECPR2 expression was reduced in the hindbrain region (Supplementary Fig. 5c). Gene expression levels in each sample were normalized to the geometric mean of 11 housekeeping genes. Out of 757 genes analyzed, 73 genes were significantly upregulated and between 51 and 29 genes were downregulated in *Tecpr2* ki/ki hindbrain and forebrain, respectively (Fig. 5a, Supplementary Fig. 5d, Supplementary Table 4). Strikingly, genes most strongly upregulated in hindbrain were those previously described for neurodegenerative disease transcriptomics signatures such as DAM. [34] These include Cts7, as the most upregulated gene, Ccl2, Cd68, CtsD, Trem2, DAP12, Lgals3, Lpl, Cd9, Fth1, ApoE and many others (Fig. 5a). In contrast, the forebrain did not show a DAM signature (Supplementary Fig. 5d). Consistent with this observation, the hindbrain NanoString analysis showed the largest overlap with the whole brain transcriptomics (Fig. 5b). Together, this suggest that microglia in the hindbrain of *Tecpr2* ki/ki mice adopt a DAM state possibly in response to the regional swollen axons while loss of the cell bodies of projecting neurons might have occurred at an earlier time point since the cortex did not show a similar prominent microglia response.

### Aberrant microglia in *Tecpr2* knock-in mice

Next, we profiled the proteome of microglia acutely isolated from *Tecpr2* ki/ki and wt/wt mice. Enrichment of microglia was similar between both samples (Supplementary Fig. 5e). Proteomic profiles of microglia revealed major differences between *Tecpr2* ki/ki and wt/wt (Fig. 5c, Supplementary Table 5). Among the significantly upregulated proteins, we identified the DAM components APOE and LGALS3, the lipid-related proteins AUP1 and PCTP as well as several endosomal/lysosomal proteins, including LAMP1, CTSS and NPC1. Unexpectedly, proteins of likely neuronal origin like MAP1A, MAP1B, MAPT, MBP, SNCA, SYN2, and KIF5C were decreased together with the autophagy proteins ATG4B, LC3A and LC3B (Fig. 5c, Supplementary Table 5). GO term analysis of the proteomics data revealed phagosome-, lysosome- and ER-to-Golgi transport-related terms increased in the absence of TECPR2 (Fig. 5d), while endocytosis, microtubule cytoskeleton organization and several neuron-associated terms were among the most decreased pathways (Fig. 5e), possibly implicating that microglia in *Tecpr2* ki/ki mice are compromised in clearing neuronal debris due to a likewise altered endolysosome compartment. This notion is supported by the fact that a number of proteins decreased in the proteome of microglia are increased in the CSF of *Tecpr2* ki/ki mice (Fig. 5f). Consistent with an altered lysosomal compartment, we observed enlarged LAMP1-positive structures in *Tecpr2* ki/ki microglia (Fig. 5g) which likely caused the increased LAMP1 signal observed in microglia in the medulla of *Tecpr2* ki/ki mice (Fig. 2d). Notably, enlarged LAMP1-positive structures were also observed in microglia-like BV2 cells lacking TECPR2 (Fig. 5h, Supplementary Fig. 5f). When primary microglia were incubated with fluorescently labeled endocytosis substrate Dextran (Dextran-Alexa Fluor 568) [35] and the lysosomal dye SiR, only *Tecpr2* ki/ki microglia showed a trend in reduced intracellular Dextran and a significant decrease in SiR-positive puncta (Fig. 5i, j). Since SiR specifically detects active Cathepsin D [36, 37], a decrease in SiR-positive puncta is indicative of less active lysosomes albeit their increase in size (Fig. 5j). The absent of overt changes in colocalization between Dextran and SiR (Fig. 5j), however, suggests that membrane fusion seems intact in *Tecpr2* ki/ki microglia.

### TECPR2 associates with HOPS and RAB5

**Fig. 6:**
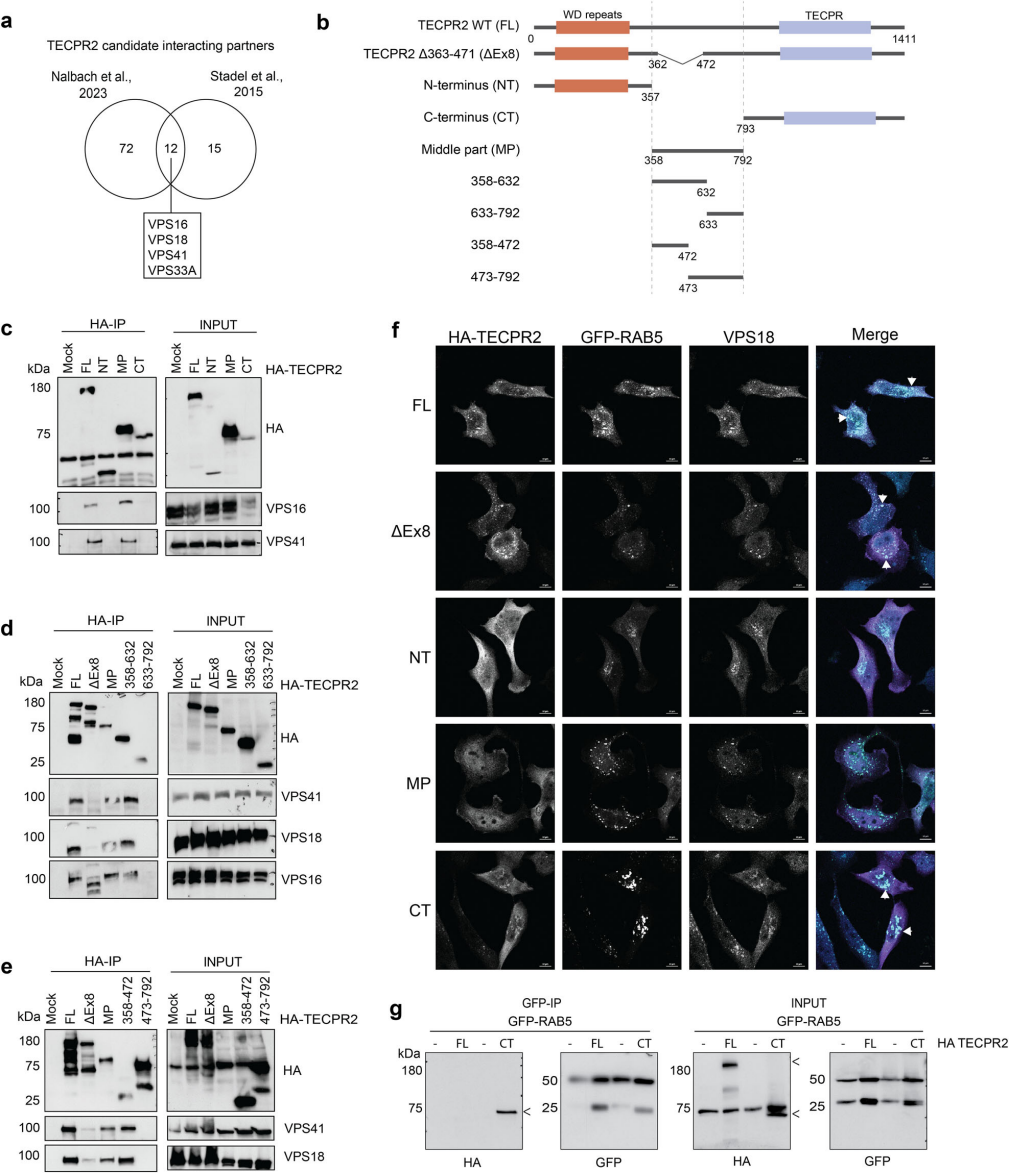
HOPS interaction with TECPR2. **a** Common TECPR2 candidate interacting proteins identified in previous interactome studies. **b** Scheme of TECPR2 fragments. **c–e** HeLa cells stably expressing indicated HA-tagged TECPR2 fragments were lysed and subjected to HA-IP followed by SDS-PAGE and immunoblotting. Empty HeLa cells (Mock) were used as control. **f** HeLa KO cells co-expressing GFP-RAB5 and indicated HA-tagged TECPR2 fragments were fixed and immunostained with anti-HA and -VPS18 antibodies. DAPI was used to stain the nucleus. Scale bar, 10 µm. **g** HeLa KO cells co-expressing GFP-RAB5 and indicated HA-tagged TECPR2 fragments were lysed and subjected to GFP-IP. < indicates specific TECPR2 bands.

Given that neuronal and microglia phenotypes converge on an altered endolysosomal system in the absence of TECPR2, we searched published TECPR2 interactomes [7, 11] for a molecular link between TECPR2 and this system. Intriguingly, the HOPS subunits VPS16, VPS18, VPS33A, and VPS41 were the only proteins found in two different proteomic studies with a clear function in the endolysosomal system (Fig. 6a). While these protein-protein interactions were biochemically validated, [7, 11] the nature of these interactions has remained largely elusive. In a first step, we sought to address whether TECPR2 binds individual HOPS subunits or the HOPS complex. For this purpose, we used biomolecular complementation affinity purification (BiCAP). Briefly, the V1 and V2 segment of the fluorescent Venus protein were fused to TECPR2 and VPS41, respectively. Following expression of V1-TECPR2 and V2-VPS41 together in HeLa cells or V2-VPS41 alone as control, we performed GFP immunoprecipitations (IPs) and analyzed interacting proteins by LC-MS (Supplementary Fig. 5g). Intriguingly, the HOPS subunits VPS16, VPS18 and VPS33A were most strongly enriched (16-fold) by TECPR2-VPS41 heterodimers (Supplementary Fig. 5h, Supplementary Table 6), suggesting that TECPR2 binds these subunits most likely as part of the HOPS complex. Next, we aimed at determining which region of TECPR2 is responsible for mediating binding to HOPS. Using an extensive panel of HA-tagged fragments covering the N-terminal WD40 repeats, the unstructured middle part and the C-terminal TECPRs (Fig. 6b), we performed HA-IPs and probed for the presence of different HOPS subunits at endogenous levels. We found that a region spanning residue 358-472 in the unstructured middle part was indispensable for binding HOPS (Fig. 6c–e). Conversely, we showed that a previously described TECPR2 mutant variant lacking residue 363-471 termed ΔEx8 [38] was deficient in binding HOPS (Fig. 6d, e). Hence, residues 363-471 seem both sufficient and necessary for HOPS binding in cell lysates. Interestingly, this region did not seem to be a general hub for interactions since other known binding partners were found to only bind full-length TECPR2 as in the case of the TRAPPIII subunit TRAPPC9 or the N-terminal and C-terminal domains as in the case of the BLOC-1 subunit DTNP1 and SNX27 (Supplementary Fig. 6a–d). To corroborate these results, we generated TECPR2 KO HeLa cells (Supplementary Fig. 6e) and monitored the subcellular distribution of HA-tagged TECPR2 variants re-expressed in these cells. Surprisingly, the presence or absence of the HOPS binding region in the unstructured middle part or in TECPR2 ΔEx8, respectively, did not correlate with TECPR2’s colocalization with the HOPS subunit VPS18 or the early endosomal GTPase RAB5 (Fig. 6f, Supplementary Fig. 6f, g). Instead, we found that the C-terminal part harboring the TECPR repeats colocalizes with VPS18 and RAB5 besides full-length TECPR2 and TECPR2 ΔEx8 (Fig. 6f, Supplementary Fig. 6f, 6g). This suggests that region 358-472 in the unstructured middle part does not drive TECPR2’s association with a HOPS-positive endosomal compartment in the cellular context. Conversely, we unveiled that the TECPR domain mediates binding to RAB5 (Fig. 6g). The fact that full-length TECPR2 did not show RAB5 binding in this assay could be explained by labile or inhibitory conformations that TECPR2 might adopt upon lysis. Thus, these findings suggest that TECPR2 requires two elements – region 358-472 and the TECPR domain – to bind HOPS and to associates with endosomal compartments [38].

### TECPR2 deficiency causes functional disturbances in the endolysosomal compartment

**Fig. 7:**
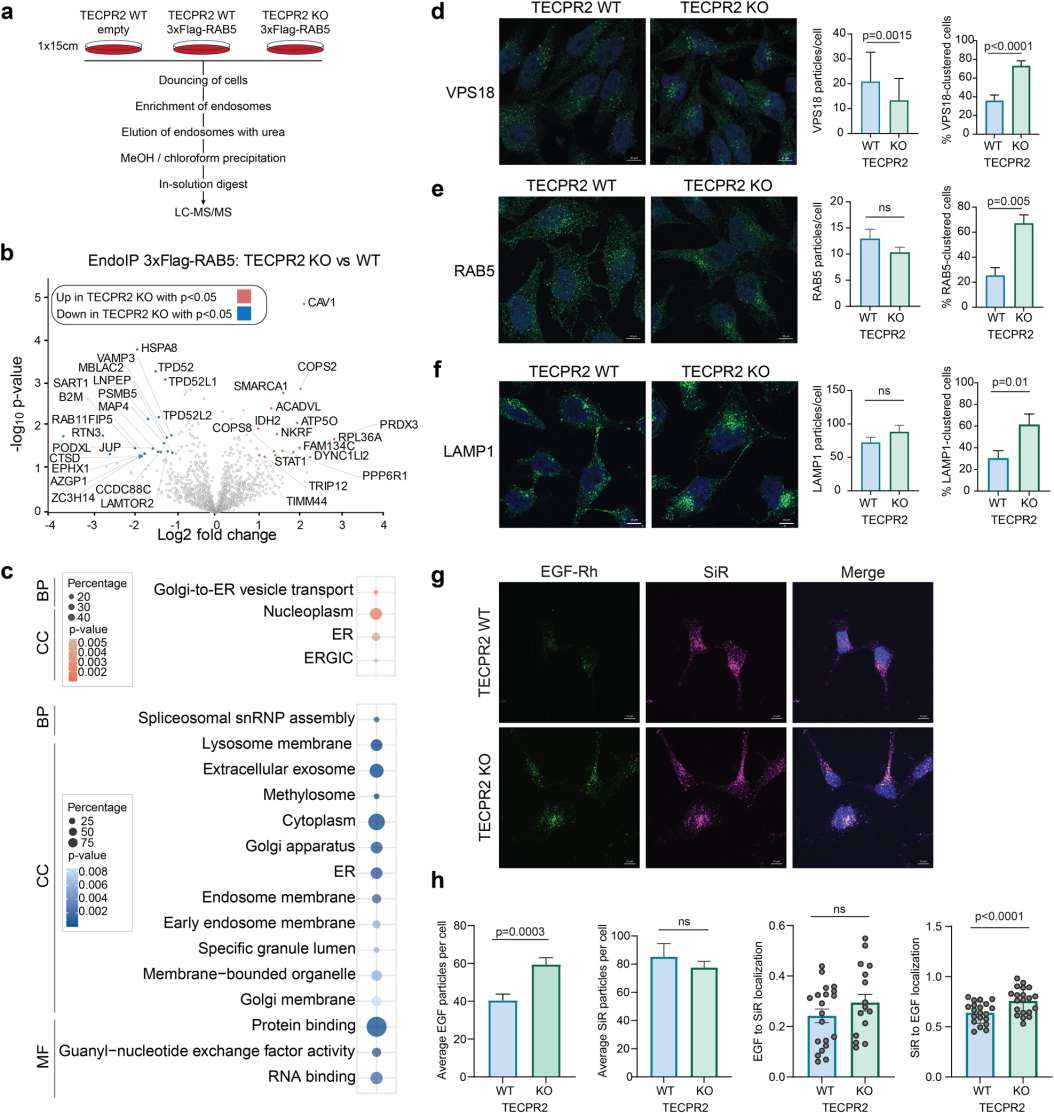
TECPR2 deficiency impacts on the endolysosomal compartment. **a** Rab5 EndoIP workflow. **b** Volcano plot representing proteins whose abundance increased- (red) or decreased (blue) in association with early endosomes enriched by Rab5 EndoIP. **c** GO terms enriched (red) or depleted (blue) in Rab5 Endo IP. **d–f** TECPR2 WT and KO cells were fixed and immunostained with indicated antibodies. DAPI was used to stain the nucleus. For particle count, 30 cells were counted over *n* = 3, for cell cluster count, >40 cells were counted over *n* = 3. Scale bar, 10 µm. **g**, **h** TECPR2 WT and KO cells were incubated with EGF-Rhodamine (Rh) and Lysosomal marker SiR particles for 1 h followed by 3 h wash-out, fixation and confocal microscopy. DAPI was used to stain the nucleus. For particle count, 30 cells were counted over *n* = 3, for co-localization, 20 cells were counted over *n* = 2 Scale bar, 10 µm. Bar graphs show mean values with SEM as error bars.

To get a better understanding of the defects in the endolysosomal system caused by TECPR2 deficiency, we performed rapid isolation of endosomes through affinity capture of the early endosome-associated protein RAB5 (EndoIP) followed by LC-MS (Fig. 7a). TECPR2 WT and KO cells were transduced with 3xFlag-RAB5A and stably overexpressed similar protein levels of this affinity handle (Supplementary Fig. 7a). Following non-detergent homogenization and anti-Flag IP, enriched endosomes from non-expressing (empty) and 3xFlag-RAB5A-expressing cells were subjected to quantitative proteomics (Fig. 7a). Notably, the former was used as negative control. EndoIP samples were substantially enriched in known endosomal and lysosomal proteins such as CD63, TFRC, LAMP1 and 2 as well as numerous v-ATPase, ESCRT and LAMTOR subunits among many others (Supplementary Fig. 7b, c, Supplementary Table 7), confirming efficient and specific capturing of endosomes. Comparison of EndoIPs from TECPR2 WT and KO cells revealed decreased abundance of proteins annotated with the GO terms endosome membrane, early endosome membrane and lysosomal membrane on endosomes from TECPR2 KO cells (Fig. 7b, c, Supplementary Table 7). Consistent with potential endolysosomal membrane alterations, we observed a significant reduction in but also clustering of puncta positive for VPS18, EEA1, or RAB7 (Fig. 7d, Supplementary Fig. 7d and 7e) without overt changes in abundance of these proteins (Supplementary Fig. 7f–i). Furthermore, increased clustering was also observed for RAB5 and LAMP1 (Fig. 7e, f). To probe the endolysosomal system for functional defects we monitored the uptake of Rhodamine (Rh)-labeled EGF and of SiR in TECPR2 WT and KO cells (Fig. 7g). Intriguingly, loss of TECPR2 led to a significant perinuclear accumulation of EGF-Rh while the number of SiR-positive structures slightly decreased (Fig. 7h). While the observed accumulation of EGF-Rh particles indicates an impairment of processing of endocytosed cargos by lysosomes, a slight decrease in SiR counts in KO cells although not significant is an indicator of less active lysosomal compartments. In addition, clustering of both endosomal and lysosomal markers around the nucleus suggests an overall block of endolysosomal compartment segregation. Collectively, this suggests that TECPR2 may play a role in endolysosomal maturation and that loss of TECPR2 may impair this process.

## Discussion

In this study, we characterize a novel mouse model generated by CRISPR knock-in of a HSAN9 disease-associated TECPR2 mutation. Although the gene encoding TECPR2 has been reported to have a number of mutations ranging from point to nonsense mutations, only a few mutations were studied in depth for their involvement in HSAN9. We confirmed in our mouse model that the nonsense mutation L1139fs leads to a complete loss of the protein evident from transcriptomics and immunoblot analysis. Behavior analysis revealed that TECPR2 ki/ki mice shows decreased anxiety, impaired sensorimotor gating, and altered gait. A broadly similar gait profile has been described in models of Parkinson’s disease (PD), spinal cord injury (SCI), and stroke [39], except for the decreased distance between the hind paws (i.e., a reduced base of support, BOS), where the opposite is typically observed in PD, and SCI. Interestingly, parallels can be drawn between the gait phenotype of the ki/ki mice and the previously characterized KO model of the same gene, as both exhibit decreased hind-paw BOS [13]. This gait phenocopy supports the presence of a shared underlying disease mechanism in these two models of deficiency.

At the histological level, ki/ki mice show clear signs of neurodegeneration, including axonal spheroids in the cuneate and gracile nuclei of the medulla oblongata in the brainstem. These regions—part of the dorsal column nuclei—are responsible for processing fine touch and proprioceptive information from the limbs and relaying it to the thalamus. The gracile nucleus transmits hind-limb sensory input, while the cuneate nucleus processes input from the forelimbs. Degeneration in these nuclei is likely to contribute to the altered gait phenotype observed. Moreover, the cuneate nucleus has been implicated in the regulation of sensorimotor gating, including prepulse inhibition, which was found to be impaired in the ki/ki mice. This deficit suggests dysfunction in sensory integration and pre-attentive processing. Given that prepulse inhibition deficits can be associated with cognitive dysfunction also in neurodevelopmental disorders [40–42], these findings may reflect aspects of the intellectual disability observed in HSAN9 patients. While direct cognitive tests were not included in this study, future work may explore this further using dedicated learning and memory paradigms.

Neuronal degeneration in the medulla seems to be primarily due to axonal swelling caused by aberrant accumulation of various organelles and proteinaceous structures ranging from swollen mitochondria, enlarged ER, lysosomes, single membrane vesicles, autophagosomes and glycogen granules as well as amorphous protein aggregates that may be composed of fragmented neurofilaments or microtubules. Importantly, the accumulation of these cellular components detected by EM together with labeling of axonal spheroids with autophagy-related proteins including LC3B and TAX1BP1, endosomal APP and presynaptic vesicle components such SYP point to defective lysosomal clearance or endolysosomal sorting in neurons lacking TECPR2.

Axonal swelling has been associated with neuronal loss in other neurodegeneration and hereditary spastic paraplegia (HSP) mice models [43–45]. For instance, HSP mice with mutations in SPG7 show dysfunctional mitochondria as the primary cause for accumulation of neurofilaments and other organelles in axonal neurites [43]. In Alzheimer’s disease (AD) mouse models, accumulation of amyloid-β-peptide (Aβ) and phosphorylated forms of Tau correlates with disorganization of microtubules in neurons leading to disturbances in anterograde trafficking of organelles [46]. Defects in autophagosome maturation or fusion to lysosomes also causes accumulation of autophagosomes in axons leading to axonal spheroid formation [45].

*Tecpr2* ki/ki mice also showed a pronounced region-specific gliosis along with loss of neurons and oligodendrocytes in the same region. Given that Tecpr2 mRNA is expressed in almost all tissues, this could indicate a composite effect of cell autonomous and non-cell autonomous roles of TECPR2. By combining transcriptomics, CSF proteomics and microglia proteome profiling, we sought to examine the consequences of TECPR2 in different brain cell types. Using this approach, we observed that along with neuronal loss there seems to be a defect in clearing of neuronal debris by microglia in Tecpr2 ki/ki mice, most likely due to reduced microglial uptake or lysosomal degradation. This is most apparent from a massive increase in neuronal proteins linked to autophagy and endocytosis in CSF and their simultaneous decrease in DAM microglia derived from *Tecpr2* ki/ki mice. Among these proteins are MAPT (Tau), γ-synuclein (SNCG), LC3B, BIN1 and adaptor protein complex 2 (AP-2) subunits. Interestingly, BIN1 plays an important role in endosomal trafficking and degradation of BACE1, an important regulator for Aβ. Decrease in BIN1 in cells has been shown to increase production and accumulation of Aβ in AD [47]. AP-2 is also involved in clathrin-mediated endocytosis of ADAM10, a protein which limits Aβ production and this process is altered in AD patients [48]. Decrease in active SiR-positive lysosomes and endocytosis of Dextran in TECPR2-deficient microglia further supports the notion that TECPR2’s role in the endolysosomal system is not restricted to neurons and that loss of TECPR2 compromises microglia’s clearing function, thereby possibly exacerbating neurodegeneration in *Tecpr2* ki/ki mice.

While TECPR2 has been reported to function in autophagy as well as in protein transport and sorting within the secretory pathway, its role in the endolysosomal system is poorly understood [7, 10, 11]. Moreover, endolysosomal defects in TECPR2 deficiency might be subsequential from disturbances in the mentioned pathways which are interconnected with the endolysosomal system. However, a direct link to the endolysosomal system was established when TECPR2 was found to interact with several subunits of HOPS, a protein complex involved in Rab5-to-Rab7 conversion, maturation of early to late endosomes and endolysosomal fusion [8]. Intriguingly, recently the HOPS component VPS41 has been reported to be mutated in patients suffering from neurodegenerative conditions such as ataxia and dystonia [12]. In addition, VPS41 was shown to have neuroprotective function against α-synuclein aggregation in PD [49]. We therefore hypothesized that TECPR2’s interaction with HOPS is important for its role in the endolysosomal system in neurons and microglia.

We unveiled that TECPR2 employs a defined region (residue 358-472) of the unstructured middle part to bind HOPS while localization to early endosomes requires interaction with RAB5 via its C-terminal TECP repeats. Notably, HOPS and the related CORVET complex are known to exist in a HOPS-CORVET hybrid complexes to transition between early and late endosomes [8, 50]. Given that the HOPS subunits VPS11 and VPS39 were neither found in previous extensive TECPR2 interactome studies nor in our current BiCAP approach, we speculate that TECPR2 might stabilize a transition form of HOPS or an alternative assembly of the HOPS subunits. A block in endolysosomal compartment segregation in TECPR2 KO cells was apparent based on the observed perinuclear clustering of early endosomal (EEA1, Rab5), late endosomal-lysosomal (RAB7, LAMP1), and HOPS (VPS18) markers. Interestingly, HOPS KO was recently shown to cause a block in endolysosomal compartment segregation and formation of HOPS bodies which are less acidic and contains less active cathepsins [37]. Given the phenotypic similarities of these KOs and the interaction of both proteins, it is conceivable that TECPR2 might be a positive regulator of HOPS whose assembly or function is compromised when TECPR2 is absent. The observation that loss of TECPR2 causes a delay in degradation of EGFR, which is known to be HOPS dependent, corroborates our hypothesis.

Overall, our study provides molecular and (patho)physiological insights into the role of TECPR2 in the endolysosomal system. While our mouse model confirms that loss of TECPR2 causes neurodegeneration, we find that other brain cell types, in particular microglia, are affected in their functioning and/or responsiveness which might in turn contribute to disease progression.

## Supplementary information

Supplementary Information

Supplementary Table Info

Supplementary Table 1

Supplementary Table 2

Supplementary Table 3

Supplementary Table 4

Supplementary Table 5

Supplementary Table 6

Supplementary Table 7

Supplementary Table 8

Uncropped Immunoblots

## Data Availability

All data are available upon reasonable request. The proteomics data have been deposited to the ProteomeXchange Consortium via the PRIDE partner repository with the dataset identifier PXD059057 (CSF), PXD065944 (Medulla) and PXD059099 (Microglia). The transcriptomics dataset were submitted to GEO under the accession number GSE284475: https://eur05.safelinks.protection.outlook.com/?url=https%3A%2F%2Fwww.ncbi.nlm.nih.gov%2Fgeo%2Fquery%2Facc.cgi%3Facc%3DGSE284475&data=05%7C02%7Cmartin.irmler%40helmholtz-munich.de%7C6f646fa5a9cf485e7ab808dd1ea2ca5f%7Ce229e4931bf240a79b8485f6c23aeed8%7C0%7C0%7C638700406722059921%7CUnknown%7CTWFpbGZsb3d8eyJFbXB0eU1hcGkiOnRydWUsIlYiOiIwLjAuMDAwMCIsIlAiOiJXaW4zMiIsIkFOIjoiTWFpbCIsIldUIjoyfQ%3D%3D%7C0%7C%7C%7C&sdata=IbNXjcV0KjbrP9%2Bi3O2%2FgeugJEBsD3apdhMZXY9JrsU%3D&reserved=0(token: mhahegkcffkftax).
