# Supplementary material for: Neuropathy-associated Tecpr2 mutation knock-in mice reveal endolysosomal loss of function phenotypes in neurons and microglia
Source: Cell Death Dis. 2025 Oct 31;16(1):775. doi: 10.1038/s41419-025-08168-w (PMC12578842; doi:10.1038/s41419-025-08168-w)
Supplement: Supplementary file 1 — Supplementary Information [file 41419_2025_8168_MOESM1_ESM.pdf]

Supplementary Information

Neuropathy-associated Tecpr2 mutation knock-in mice reveal endolysosomal loss of function phenotypes in neurons and microglia

Debjani Bhattacharya [1,#], Patricia da Silva-Buttkus [2,#], Karsten Nalbach [3,4], Lizhen Cheng [1], Lillian Garrett [2], Martin Irmler [2], Georg Kislinger [3,5], Georg Werner [6], Ramona Rodde [6], Christoph Lengger [2], Johannes Beckers [2], Annemarie Zimprich [2,7], Sabine M. Hölter [2], Valerie Gailus-Durner [2], Helmut Fuchs [2], Martin Hrabe de Angelis [2,8,9], Benedikt Wefers [3,10], Wolfgang Wurst [3,10,11], Monika S. Brill [5,11], Martina Schifferer [3,11], Stefan F. Lichtenthaler [3,4,11], Christian Behrends [1,12,*]

[1] Munich Cluster for Systems Neurology (SyNergy), Faculty of Medicine, Ludwig-Maximilians-Universität München, Munich, Germany

[2] Institute of Experimental Genetics and German Mouse Clinic, Helmholtz Zentrum München, German Research Center for Environmental Health, Neuherberg, Germany

[3] German Center for Neurodegenerative Diseases (DZNE), Munich, Germany

[4] Neuroproteomics, School of Medicine and Health, Klinikum rechts der Isar, Technical University of Munich, Munich, Germany

[5] Institute of Neuronal Cell Biology, Technical University of Munich, Munich, Germany

[6] Metabolic Biochemistry, Biomedical Center (BMC), Faculty of Medicine, Ludwig-Maximilians-Universität München, Munich, Germany

[7] German Center for Mental Health (DZPG), Munich site, Germany

[8] Chair of Experimental Genetics, TUM School of Life Sciences, Technische Universität München, Freising, Germany

[9] German Center for Diabetes Research (DZD), Neuherberg, Germany

[10] Institute of Developmental Genetics, Helmholtz Zentrum München, Munich Germany

[11] Munich Cluster for Systems Neurology (SyNergy), Munich, Germany

[12] Lead contact

[#] These authors contribute equally to this work.

* Correspondence to: christian.behrends@mail03.med.uni-muenchen.de

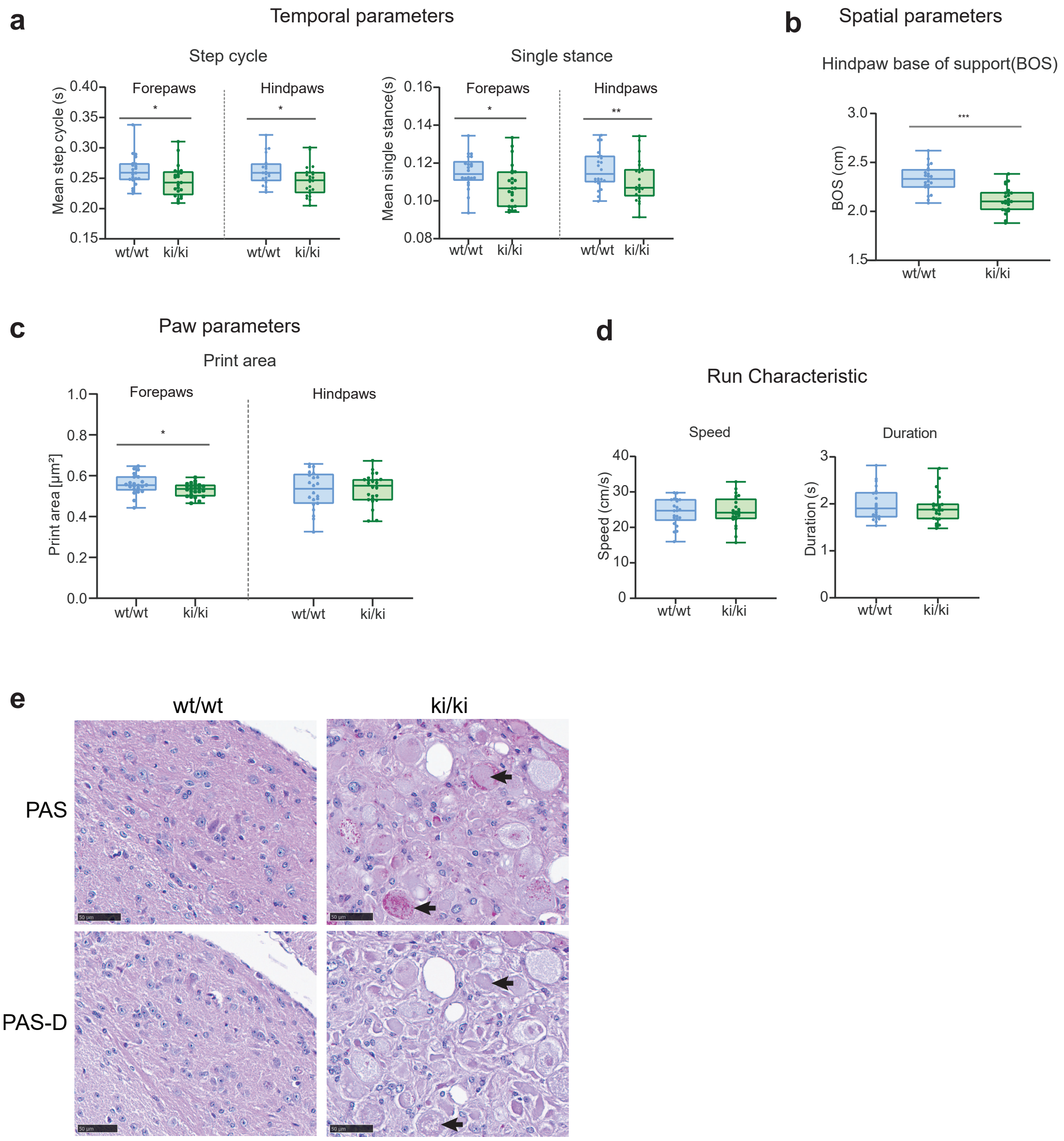
a
Temporal parameters
Step cycle
Forepaws
Hindpaws
Mean step cycle (s)
0.40
0.35
0.30
0.25
0.20
0.15
wt/wt
ki/ki
wt/wt
ki/ki
*
*
Single stance
Forepaws
Hindpaws
Mean single stance(s)
0.16
0.14
0.12
0.10
0.08
wt/wt
ki/ki
wt/wt
ki/ki
*
**
b
Spatial parameters
Hindpaw base of support(BOS)
BOS (cm)
3.0
2.5
2.0
1.5
wt/wt
ki/ki
***
c
Paw parameters
Print area
Forepaws
Hindpaws
Print area [µm²]
1.0
0.8
0.6
0.4
0.2
0.0
*
wt/wt
ki/ki
wt/wt
ki/ki
d
Run Characteristic
Speed
Speed (cm/s)
40
30
20
10
0
wt/wt
ki/ki
Duration
Duration (s)
3
2
1
0
wt/wt
ki/ki
e
wt/wt
ki/ki
PAS
PAS-D

Supplementary Figure 1. ***Tecpr2*** **ki/ki mouse behavioral studies and histology.**

a Temporal Catwalk parameter of step cycle and single stance in 5-month-old mice (10 male wt/wt, 12 female wt/wt, 12 male ki/ki, 12 female ki/ki, pooled). **b** Paw parameter of print area from mice analyzed in (c). **c** Spatial parameter of hindpaw base of support (BOS) from mice analyzed in (c). **d** Catwalk run characteristics of speed and duration from mice analyzed in (c). **e** Periodic-Acid-Schiff (PAS) and PAS-Diastase staining of brain sections from 5-month-old *Tecpr2* ki/ki and wt/wt mice. Arrow marks PAS positive structures in dark pink and the respective glycogen digested structures upon diastase treatment. Scale bar: 50 µm

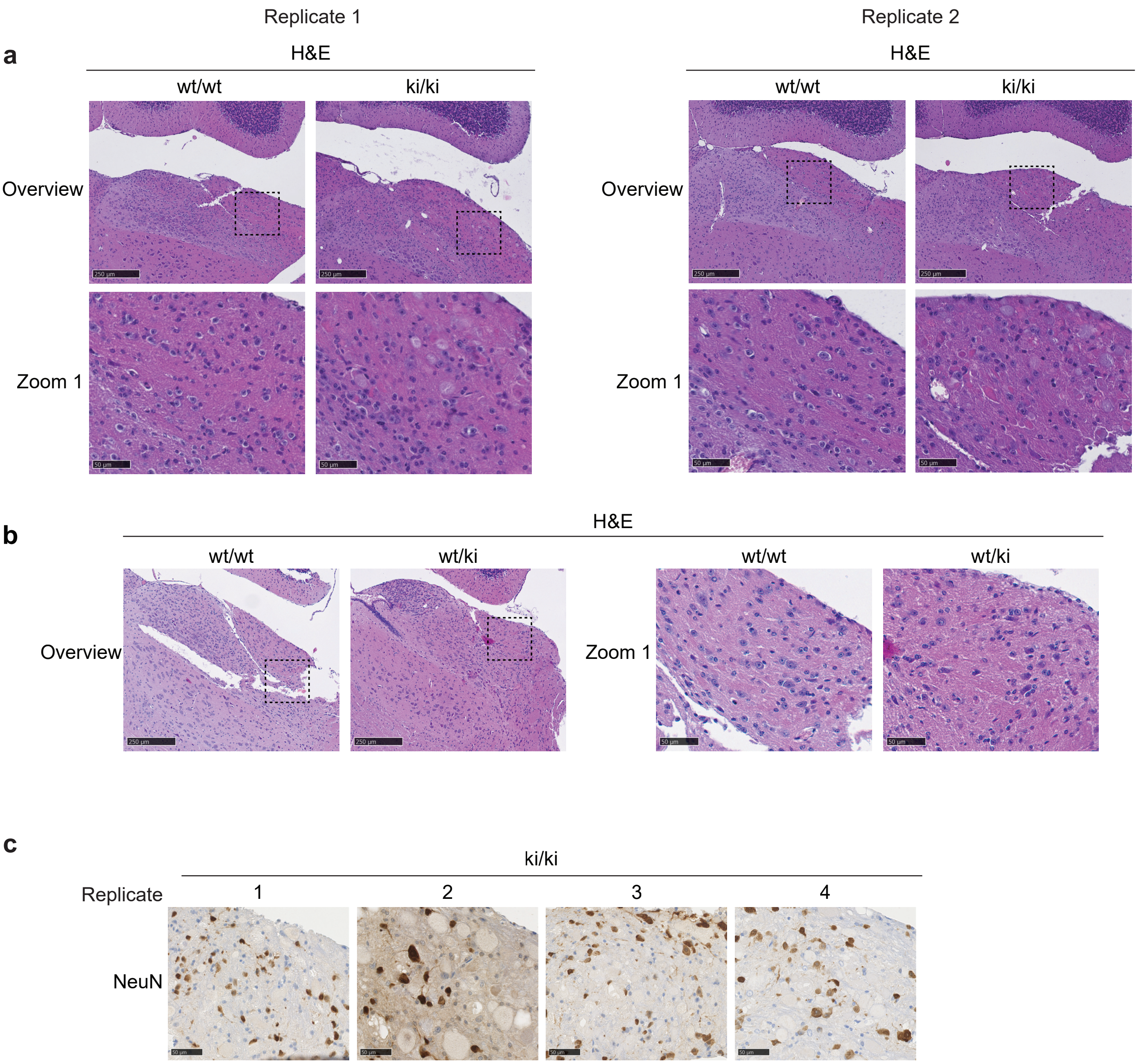

Replicate 1
Replicate 2
a
H&E
H&E
wt/wt
ki/ki
wt/wt
ki/ki
Overview
Overview
Zoom 1
Zoom 1
b
H&E
wt/wt
wt/ki
wt/wt
wt/ki
Overview
Zoom 1
c
ki/ki
Replicate
1
2
3
4
NeuN

Supplementary Figure 2. Histology and immunohistochemistry of *Tecpr2* ki/ki mouse brainstem area from 2- and 5-months old *Tecpr2* ki/ki mice.

a Hematoxylin and eosin staining of representative paraffin embedded brain sections from 2 replicates of 2-month-old *Tecpr2* ki/ki and wt/wt mice (n=2). Boxes show affected brain stem region. Scale bar: 250 µm (overview), 50 µm (zoom). **b** Hematoxylin and eosin staining of representative paraffin embedded brain sections from of 2-month-old *Tecpr2* wt/ki and wt/wt mice. Boxes show zoom 1 of brain stem region. Scale bar: 250 µm (overview), 50 µm (zoom). **c** Representative images of immunohistochemical staining of NeuN in the medulla of 4 individual 5-months old *Tecpr2* ki/ki mice (n=10). Scale bar: 50 µm.

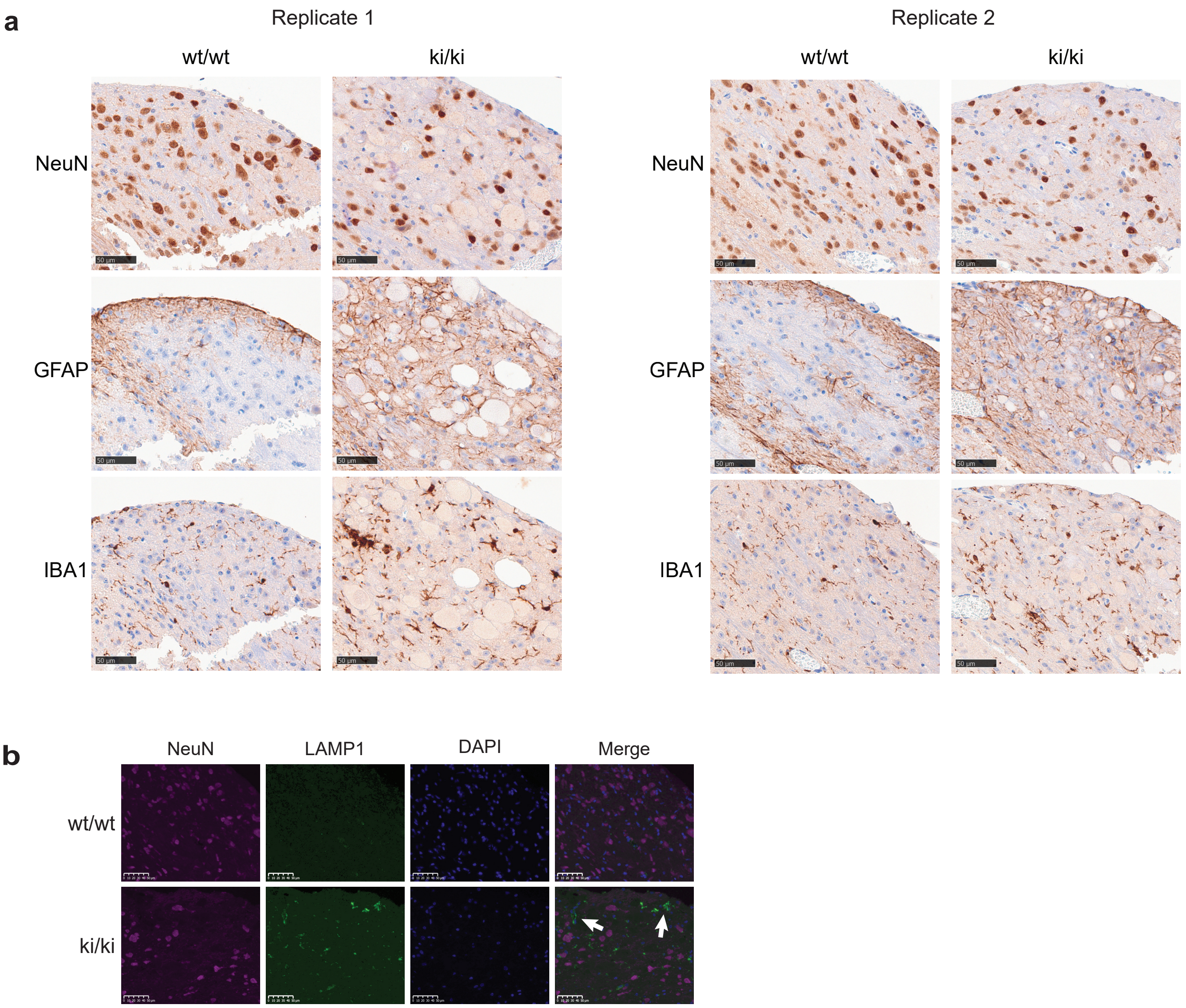
a
Replicate 1
wt/wt
ki/ki
Replicate 2
wt/wt
ki/ki
NeuN
GFAP
IBA1
NeuN
GFAP
IBA1
b
NeuN
LAMP1
DAPI
Merge
wt/wt
ki/ki

Supplementary Figure 3. Immunohistological and immunofluorescent analysis of 2- and 5-months old *Tecpr2* ki/ki mouse brainstem area.

a Immunohistochemical staining of NeuN, GFAP and IBA1 from 2 replicates of 2-month-old Tecpr2 ki/ki and wt/wt mice (n=2). Scale bar: 50 µm **b** Colocalization analysis of NeuN and LAMP1 in medulla of *Tecpr2* ki/ki and wt/wt mice. Arrows indicate that increased LAMP1 signal does not colocalize with NeuN. Scale bar: 50 µm

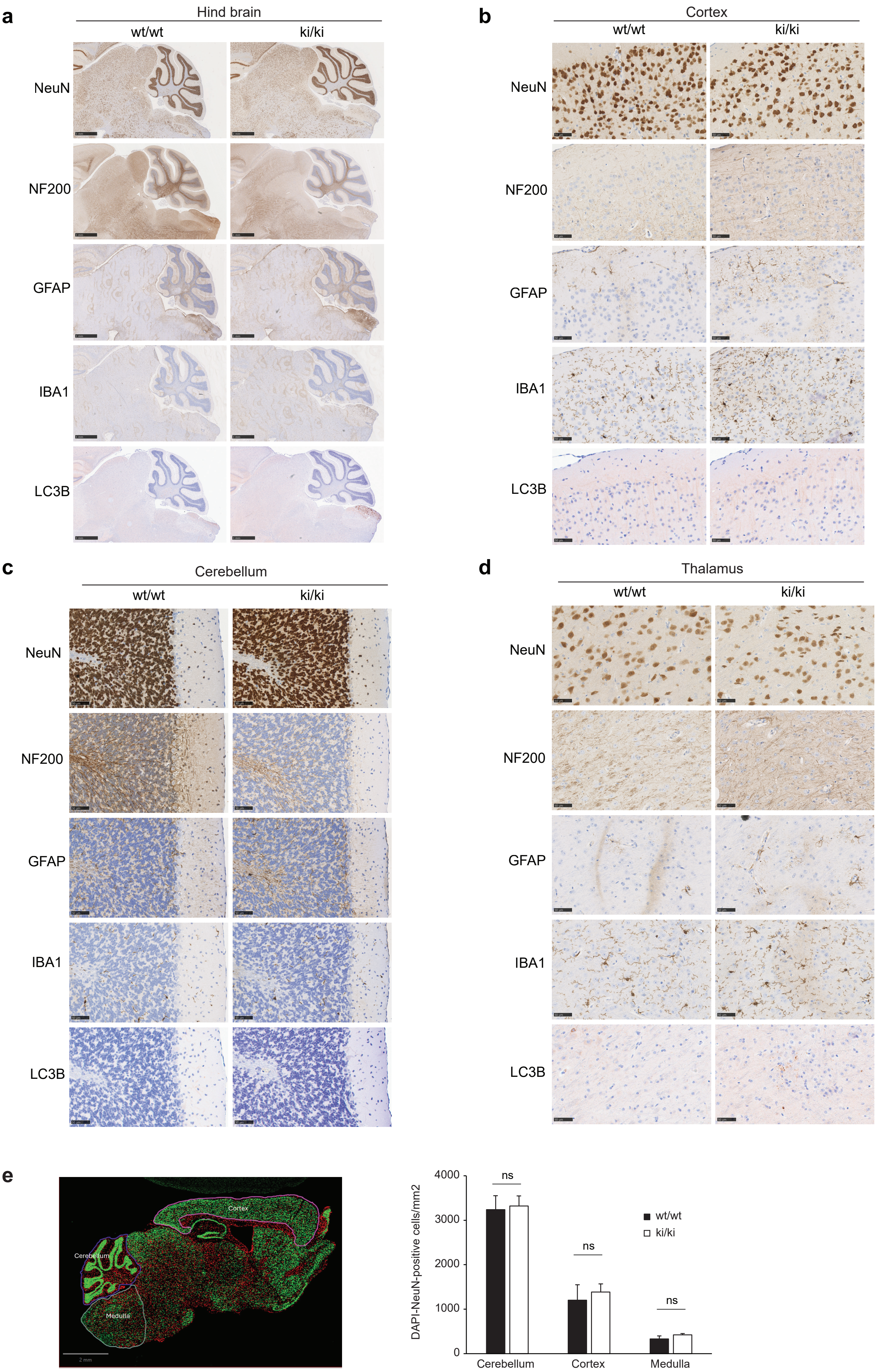

a
Hind brain
wt/wt
ki/ki
NeuN
NF200
GFAP
IBA1
LC3B
b
Cortex
wt/wt
ki/ki
NeuN
NF200
GFAP
IBA1
LC3B
c
Cerebellum
wt/wt
ki/ki
NeuN
NF200
GFAP
IBA1
LC3B
d
Thalamus
wt/wt
ki/ki
NeuN
NF200
GFAP
IBA1
LC3B
e
Cortex
Cerebellum
Medulla
2 mm
DAPI-NeuN-positive cells/mm2
4000
3000
2000
1000
0
ns
ns
ns
wt/wt
ki/ki
Cerebellum
Cortex
Medulla

Supplementary Figure 4. Whole brain immunohistology of *Tecpr2* ki/ki mice.

a Immunohistochemical stainings with indicated neuronal, glial and autophagosomal markers of the hindbrain area of 5-month-old *Tecpr2* ki/ki mice. Age-matched wildtype littermates were used as control (n=10). Scale bar: 1 mm. **b** Immunostaining of neuronal, glial and autophagosomal markers in the cortex of 5-month-old *Tecpr2* ki/ki and wt/wt mice (n=10). Scale bar: 50 µm. **c** Immunostaining of neuronal, glial and autophagosomal markers in the cerebellum of 5-month-old *Tecpr2* ki/ki and wt/wt mice (n=10). Scale bar: 50 µm **d** Immunostaining of neuronal, glial and autophagosomal markers in the thalamus of 5-month-old *Tecpr2* ki/ki and wt/wt mice (n=10). Scale bar: 50 µm **e** Quantitative analysis of NeuN-positive cells per mm^2 in the cortex, medulla and cerebellum of *Tecpr2* ki/ki and wt/wt mice (n=4/group).

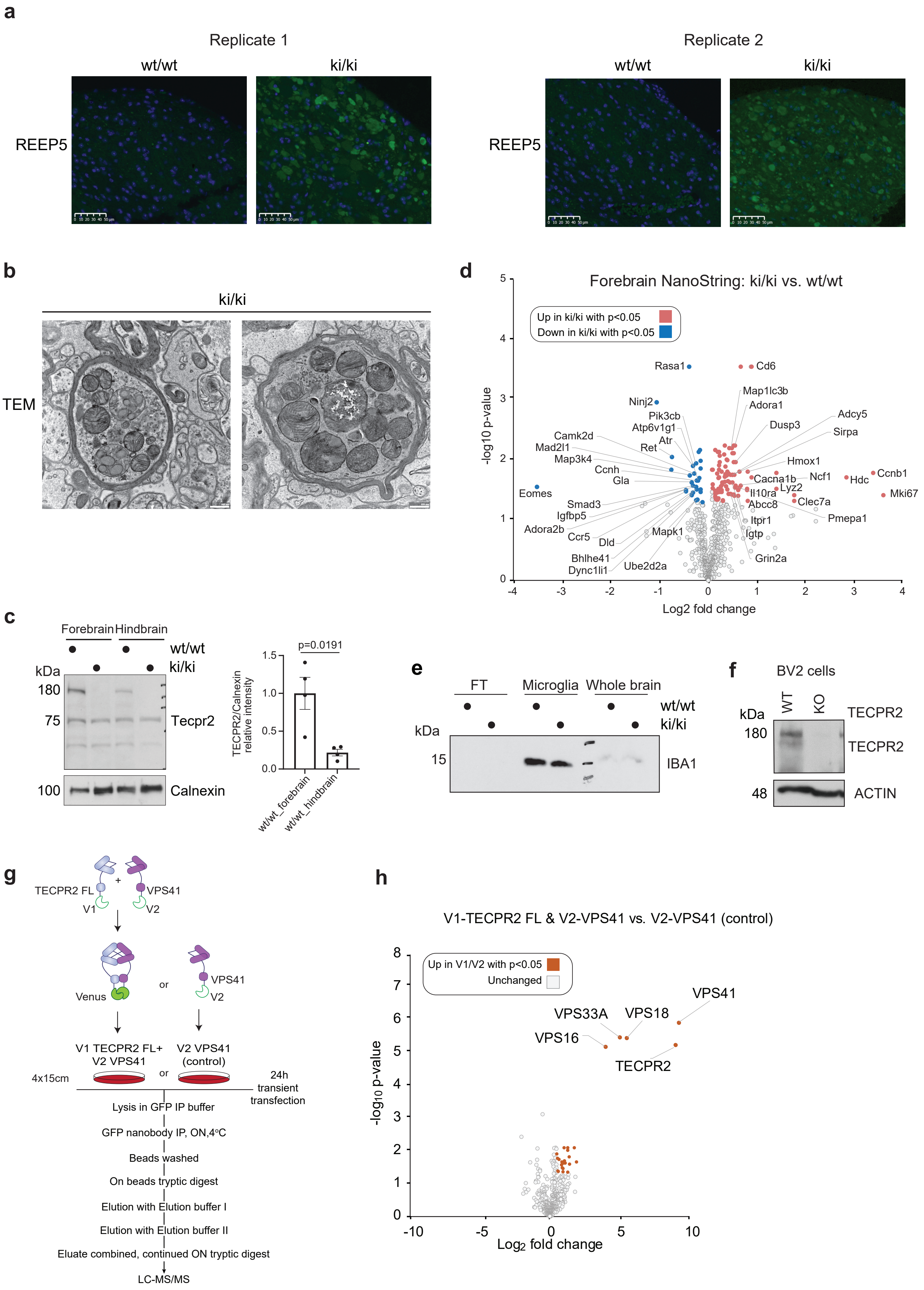

a
Replicate 1
wt/wt
ki/ki
REEP5
Replicate 2
wt/wt
ki/ki
REEP5
b
ki/ki
TEM
c
Forebrain
Hindbrain
wt/wt
ki/ki
kDa
180
75
Tecpr2
100
Calnexin
p=0.0191
TECPR2/Calnexin relative intensity
1.5
1.0
0.5
0.0
wt/wt_forebrain
wt/wt_hindbrain
d
Forebrain NanoString: ki/ki vs. wt/wt
Up in ki/ki with p<0.05
Down in ki/ki with p<0.05
-log10 p-value
Log2 fold change
Rasa1
Cd6
Map1lc3b
Adora1
Ninj2
Pik3cb
Atp6v1g1
Dusp3
Adcy5
Sirpa
Camk2d
Mad2l1
Map3k4
Ccnh
Gla
Atr
Ret
Hmox1
Cacna1b
Ncf1
Hdc
Ccnb1
Il10ra
Lyz2
Mki67
Eomes
Smad3
Igfbp5
Adora2b
Ccr5
Dld
Bhlhe41
Dync1li1
Mapk1
Ube2d2a
Abcc8
Clec7a
Itpr1
Igtp
Pmepa1
Grin2a
e
FT
Microglia
Whole brain
wt/wt
ki/ki
kDa
15
IBA1
f
BV2 cells
WT
KO
kDa
180
48
TECPR2
TECPR2
ACTIN
g
TECPR2 FL
VPS41
V1
V2
Venus
or
VPS41
V2
V1 TECPR2 FL+ V2 VPS41
V2 VPS41 (control)
4x15cm
or
24h transient transfection
Lysis in GFP IP buffer
GFP nanobody IP, ON,4°C
Beads washed
On beads tryptic digest
Elution with Elution buffer I
Elution with Elution buffer II
Eluate combined, continued ON tryptic digest
LC-MS/MS
h
V1-TECPR2 FL & V2-VPS41 vs. V2-VPS41 (control)
Up in V1/V2 with p<0.05
Unchanged
VPS41
VPS33A
VPS18
VPS16
TECPR2
-log10 p-value
Log2 fold change

Supplementary Figure 5. Endoplasmic reticulum staining in TECPR2 *Tecpr2* ki/ki mouse brain.

a Immunofluorescence staining of ER marker REEP5 in the medulla of representative paraffin embedded brain sections from 2 replicates of 2-month-old *Tecpr2* ki/ki and wt/wt mice (n=2). **b** Transmission electron microscopy (TEM) images from samples shown in Figure 4f depicting higher resolutions of accumulated aberrant mitochondria in axons. Scale bar: 500 nm. **c** Immunoblot comparison of TECPR2 protein level in forebrain versus hindbrain region of *Tecpr2* wt/wt. (n=3) **d** Volcano plot of up- or downregulated genes detected by NanoString analysis in the forebrain of 12-month-old *Tecpr2* ki/ki and wt/wt mice (n = 6, males). Genes with $-Log_{10}$(p-value) ≥1.3 are highlighted in red (up) and blue (down). Top20 up- and downregulated genes are indicated. **e** Immunoblot analysis of microglia enrichment from 12-month-old *Tecpr2* ki/ki mice. FT, flow through. **f** Immunoblot analysis validating TECPR2 Knock-out in BV2 cell line. **g** BiCAP scheme and workflow. **h** Volcano plot depicting protein identified by BiCAP of V1-TECPR2 and V2-VPS41. V2-VPS41 alone was used as negative control. Bar graphs show mean values with SEM as error bars.

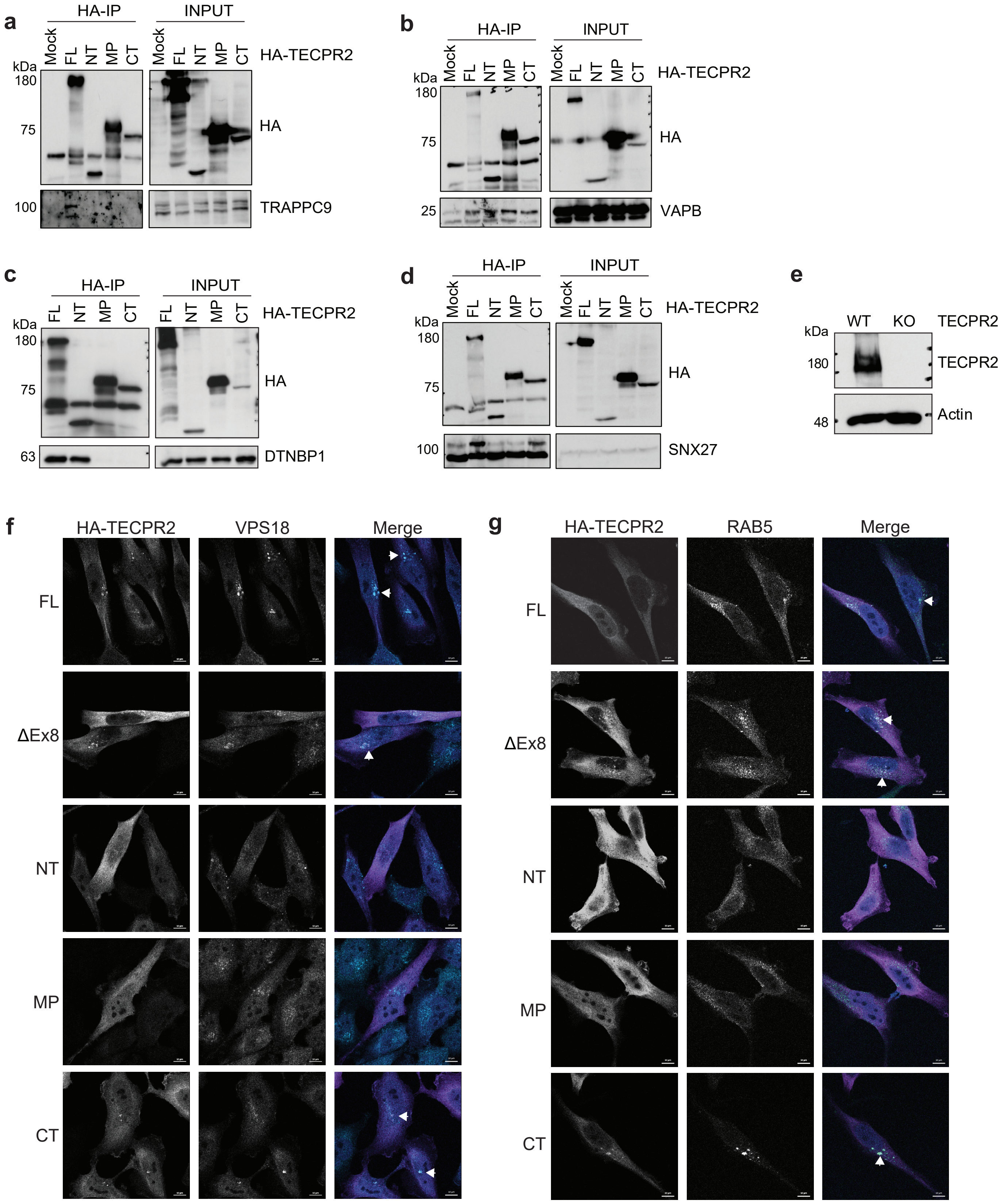
a
HA-IP
INPUT
Mock
FL
NT
MP
CT
HA-TECPR2
kDa
180
75
100
HA
TRAPPC9
b
HA-IP
INPUT
HA-TECPR2
180
75
25
HA
VAPB
c
HA-IP
INPUT
HA-TECPR2
180
75
63
HA
DTNBP1
d
HA-IP
INPUT
HA-TECPR2
180
75
100
HA
SNX27
e
WT
KO
TECPR2
kDa
180
48
TECPR2
Actin
f
HA-TECPR2
VPS18
Merge
FL
ΔEx8
NT
MP
CT
g
HA-TECPR2
RAB5
Merge
FL
ΔEx8
NT
MP
CT

Supplementary Figure 6. HOPS-interacting region in TECPR2.

a-d HeLa cells stably expressing indicated HA-tagged TECPR2 fragments were lysed and subjected to HA-IP followed by SDS-PAGE and immunoblotting. Empty HeLa cells (Mock) were used as control. **e,f** HeLa cells expressing indicated HA-tagged TECPR2 fragments were fixed and immunostained with anti-VPS18 (e) and -RAB5 (f) antibodies. DAPI was used to stain the nucleus. Scale bar, 10 μm.

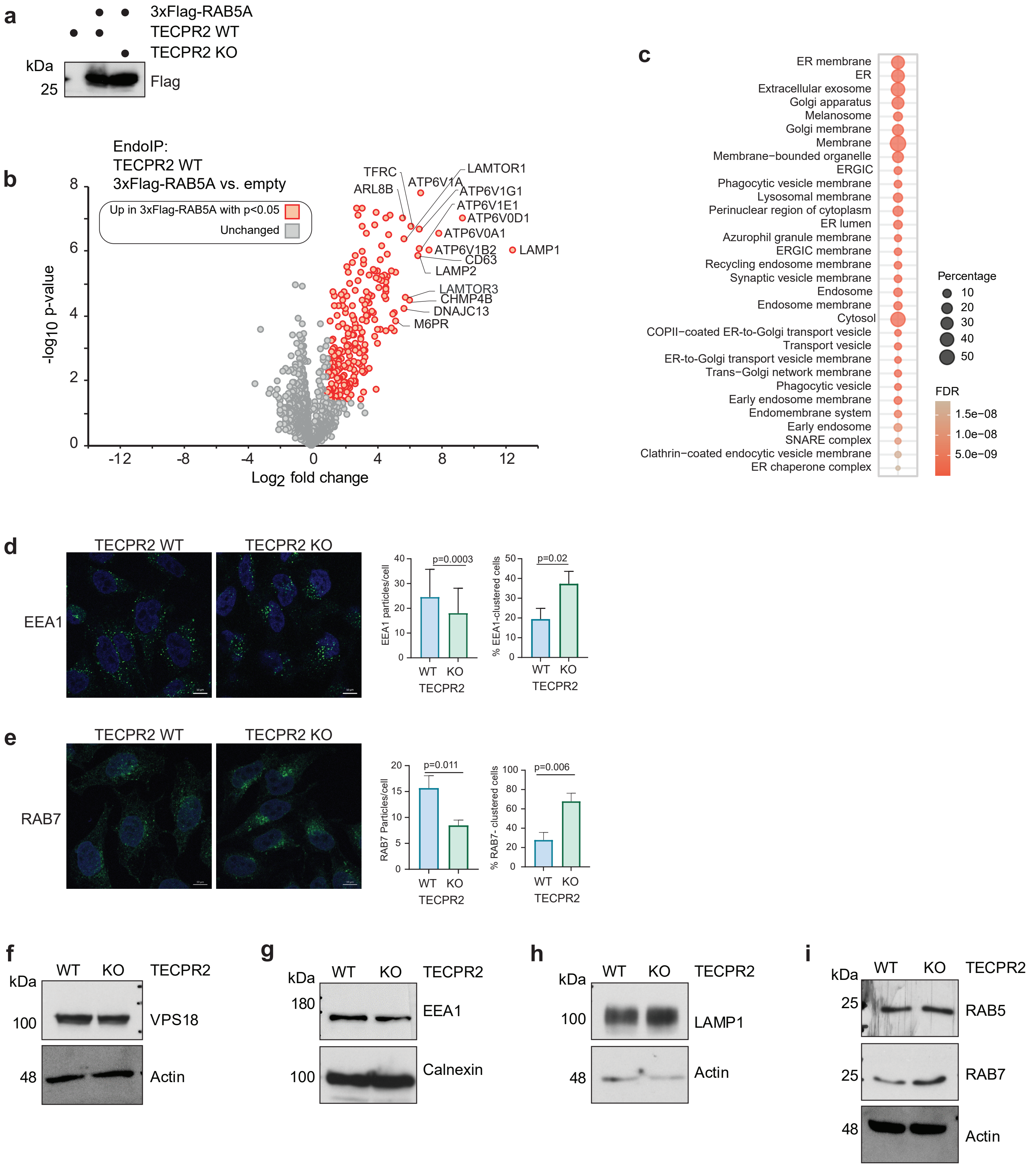
a
3xFlag-RAB5A
TECPR2 WT
TECPR2 KO
kDa
25
Flag
b
EndoIP:
TECPR2 WT
3xFlag-RAB5A vs. empty
Up in 3xFlag-RAB5A with p<0.05
Unchanged
-log10 p-value
Log2 fold change
TFRC
ARL8B
ATP6V1A
LAMTOR1
ATP6V1G1
ATP6V1E1
ATP6V0D1
ATP6V0A1
ATP6V1B2
LAMP1
CD63
LAMP2
LAMTOR3
CHMP4B
DNAJC13
M6PR
c
ER membrane
ER
Extracellular exosome
Golgi apparatus
Melanosome
Golgi membrane
Membrane
Membrane−bounded organelle
ERGIC
Phagocytic vesicle membrane
Lysosomal membrane
Perinuclear region of cytoplasm
ER lumen
Azurophil granule membrane
ERGIC membrane
Recycling endosome membrane
Synaptic vesicle membrane
Endosome
Endosome membrane
Cytosol
COPII−coated ER-to-Golgi transport vesicle
Transport vesicle
ER-to-Golgi transport vesicle membrane
Trans−Golgi network membrane
Phagocytic vesicle
Early endosome membrane
Endomembrane system
Early endosome
SNARE complex
Clathrin−coated endocytic vesicle membrane
ER chaperone complex
Percentage
10
20
30
40
50
FDR
1.5e−08
1.0e−08
5.0e−09
d
TECPR2 WT
TECPR2 KO
EEA1
EEA1 particles/cell
p=0.0003
% EEA1-clustered cells
p=0.02
WT KO
TECPR2
e
TECPR2 WT
TECPR2 KO
RAB7
RAB7 Particles/cell
p=0.011
% RAB7- clustered cells
p=0.006
WT KO
TECPR2
f
WT KO TECPR2
kDa
100
VPS18
48
Actin
g
WT KO TECPR2
kDa
180
EEA1
100
Calnexin
h
WT KO TECPR2
kDa
100
LAMP1
48
Actin
i
WT KO TECPR2
kDa
25
RAB5
25
RAB7
48
Actin

Supplementary Figure 7. TECPR2 deficiency impacts on the endolysosomal compartment.

a Validation of EndoIP cell lines. **b** GO terms enriched in EndoIP in TECPR2 WT cells. Empty TECPR2 WT cells were used as control. **c,d** TECPR2 WT and KO cells were fixed and immunostained with anti-CD63 (c) or -RAB7 (d) antibodies. DAPI was used to stain the nucleus. Scale bar, 10 µm. For particle count, 30 cells were counted over 3n, for cell cluster count, >40 cells were counted over 3n. **e-j** TECPR2 WT and KO cells were lysed and subjected to SDS-PAGE and immunoblotting with indicated antibodies. Bar graphs show mean values with SEM as error bars.
