# Supplementary material for: Neuropathy-associated Tecpr2 mutation knock-in mice reveal endolysosomal loss of function phenotypes in neurons and microglia
Source: Cell Death Dis. 2025 Oct 31;16(1):775. doi: 10.1038/s41419-025-08168-w (PMC12578842; doi:10.1038/s41419-025-08168-w)
Supplement: Supplementary file 11 — Uncropped Immunoblots [file 41419_2025_8168_MOESM11_ESM.pdf]

1. Uncropped Western blots for figure 1b

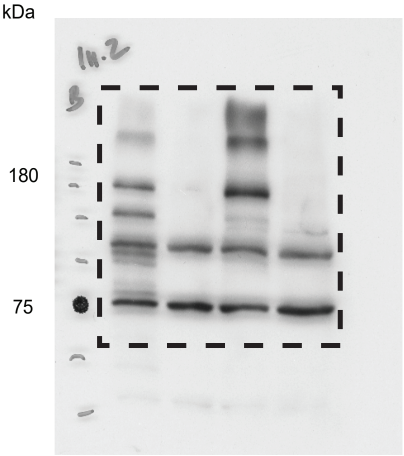

IB: TECPR2

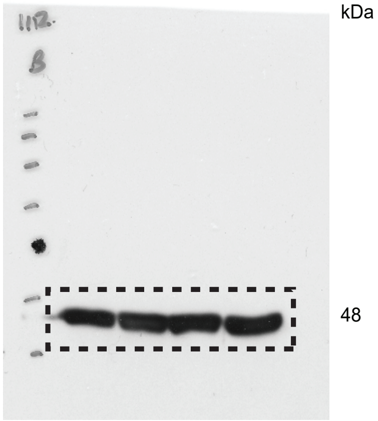

IB: TUBULIN

2. Uncropped Western blots for figure 6c

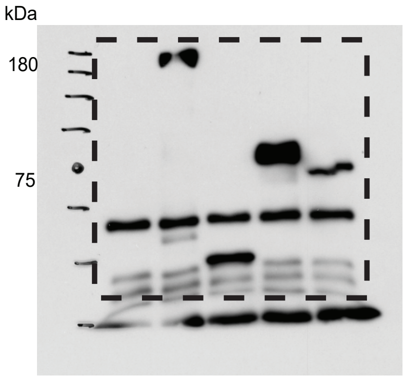

IB:HA-IP

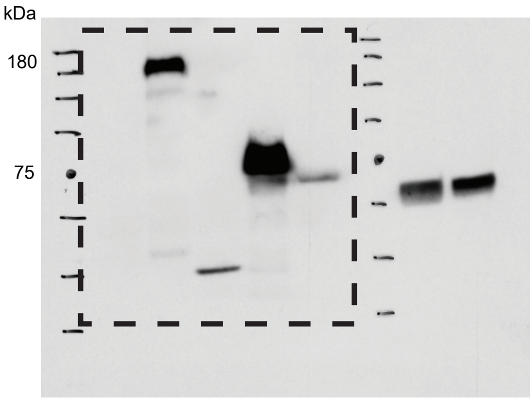

IB:HA-INPUT

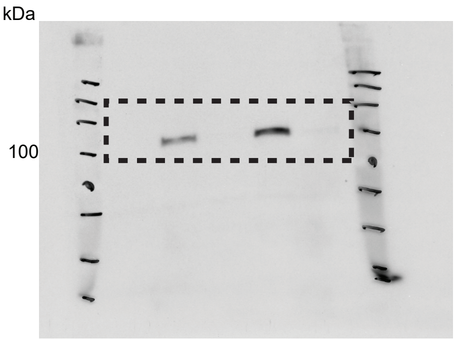

IB:VPS16-IP

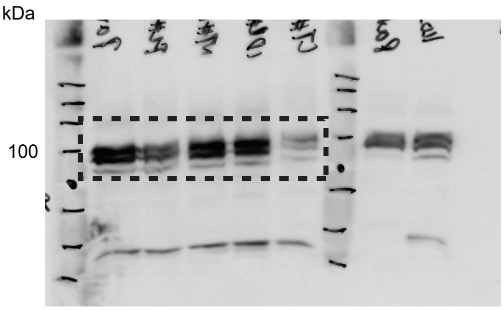

IB:VPS16-INPUT

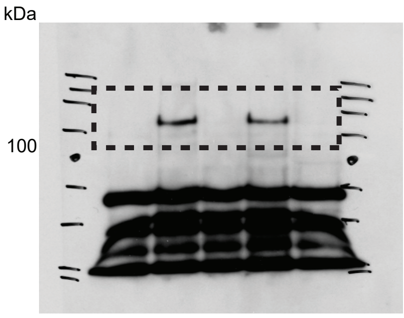

IB:VPS41-IP

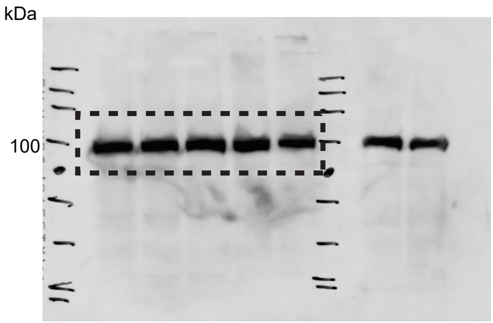

IB:VPS41-INPUT

3. Uncropped Western blots for figure 6d

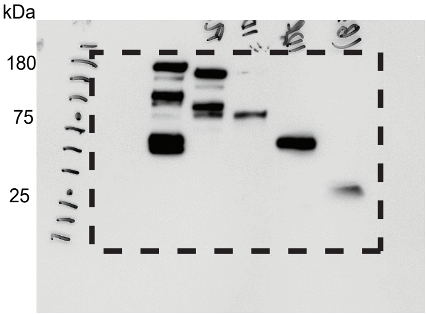

IB:HA-IP

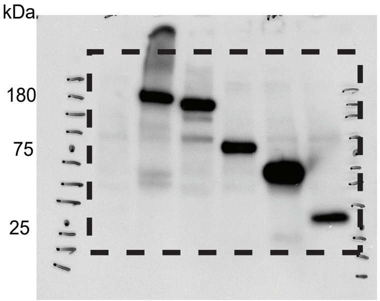

IB:HA-INPUT

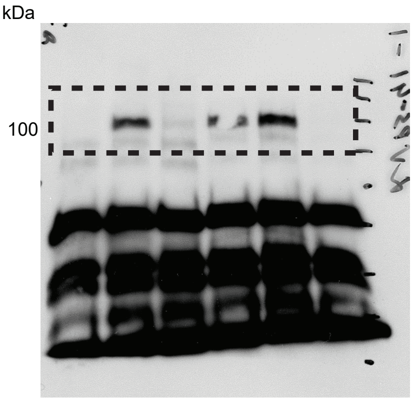

IB:VPS41-IP

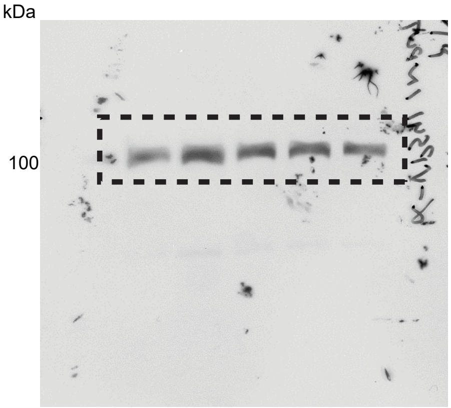

IB:VPS41-INPUT

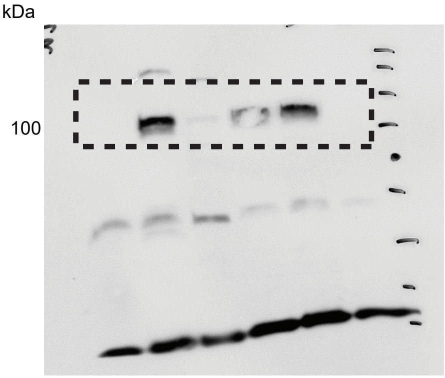

IB:VPS18-IP

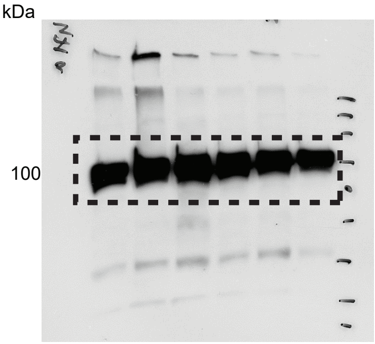

IB:VPS18-INPUT

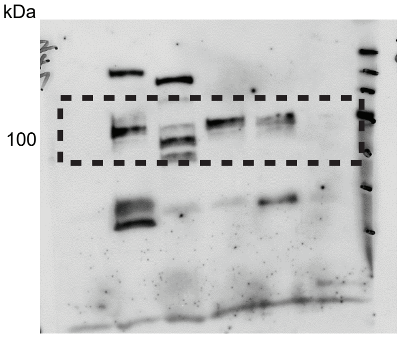

IB:VPS16-IP

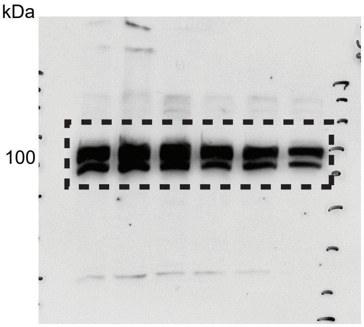

IB:VPS16-INPUT

4. Uncropped Western blots for figure 6e

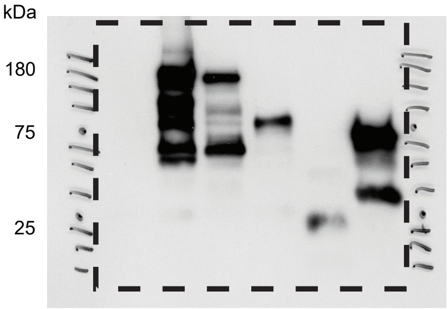

IB:HA-IP

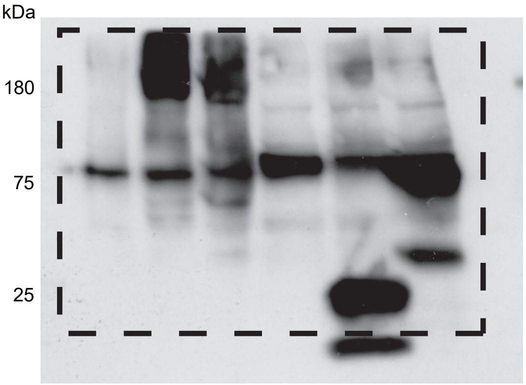

IB:HA-INPUT

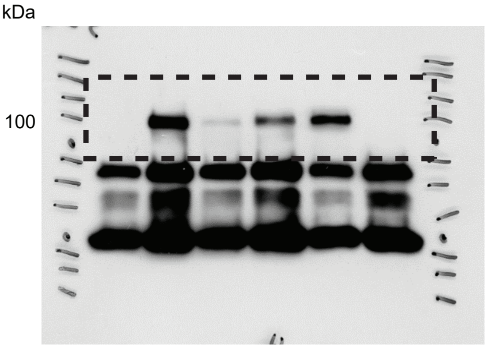

IB:VPS41-IP

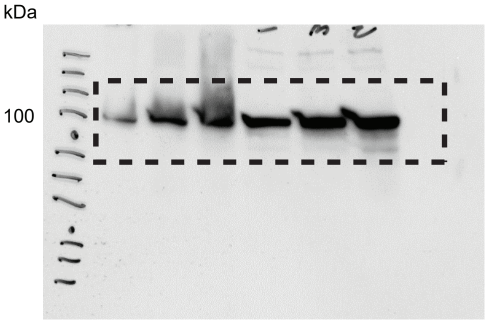

IB:VPS41-INPUT

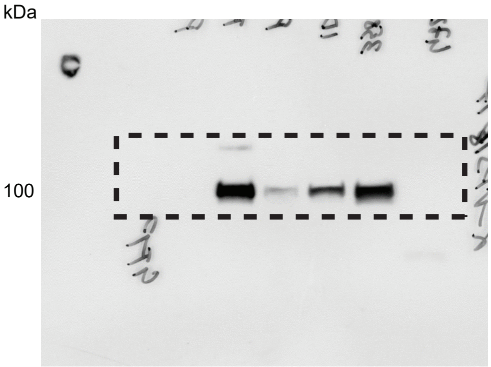

IB:VPS18-IP

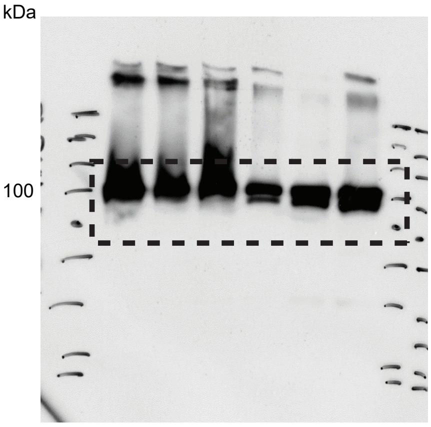

IB:VPS18-INPUT

5. Uncropped Western blots for figure 6f

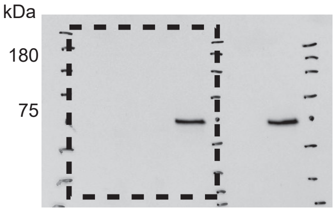

IB:HA-IP

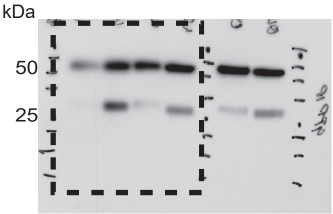

IB:GFP-IP

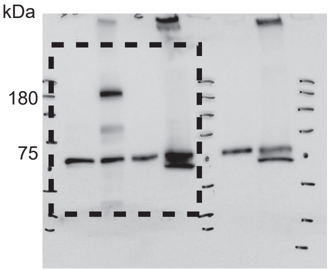

IB:HA-INPUT

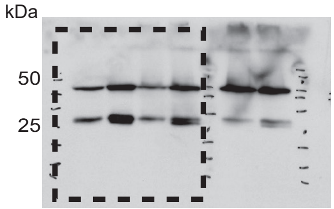

IB:GFP-INPUT

6. Uncropped Western blots for Supplementary figure 5b

kDa
180

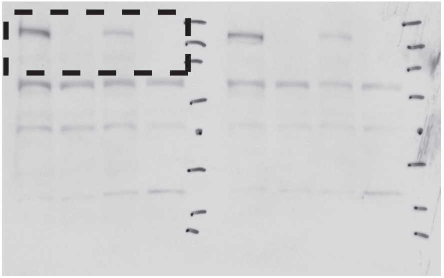

IB:TECPR2

kDa
75

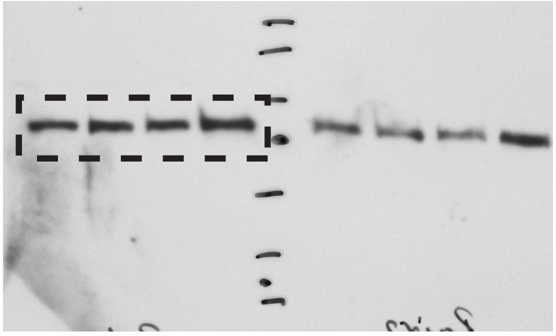

IB:Calnexin

7. Uncropped Western blots for Supplementary figure 5d

kDa
15

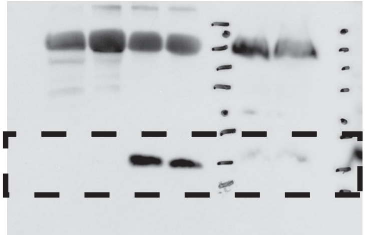

IB:IBA1

8. Uncropped Western blots for Supplementary figure 5e

kDa
180

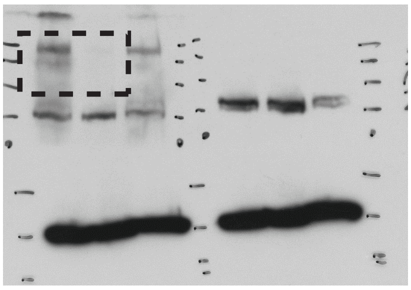

IB:TECPR2

kDa
48

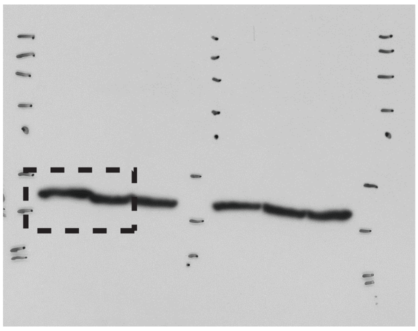

IB:Actin

9. Uncropped Western blots for Supplementary figure 6a

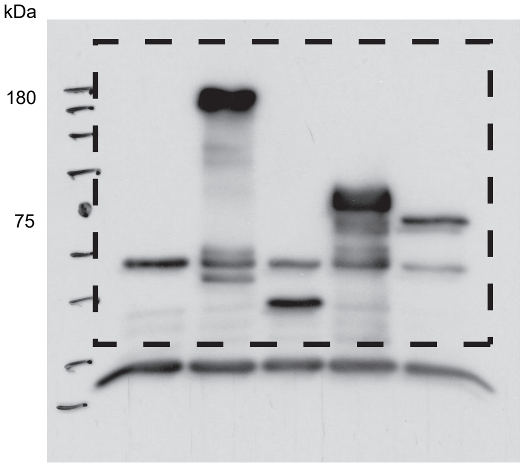

IB:HA-IP

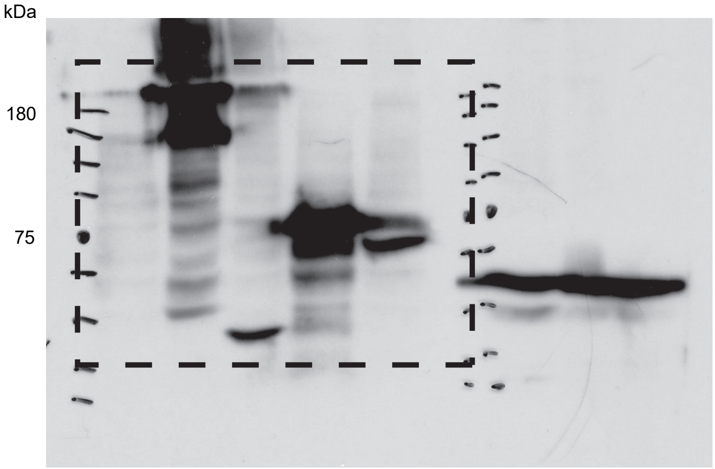

IB:HA-INPUT

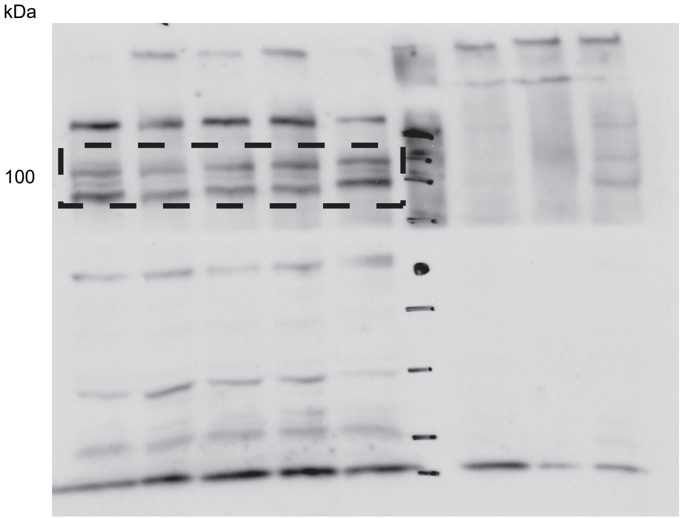

IB:TRAPPC9-IP

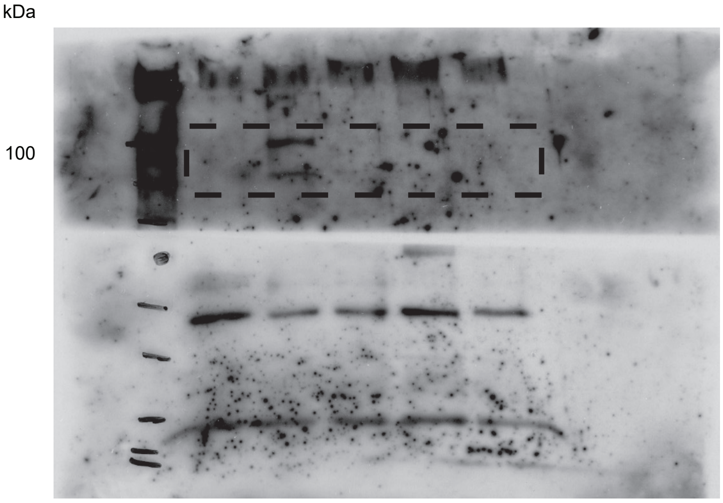

IB:TRAPPC9-INPUT

10. Uncropped Western blots for Supplementary figure 6b

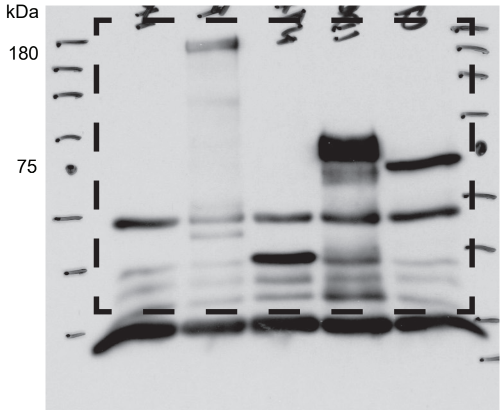

IB:HA-IP

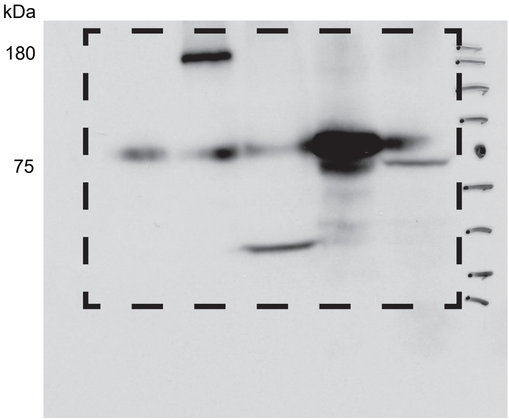

IB:HA-INPUT

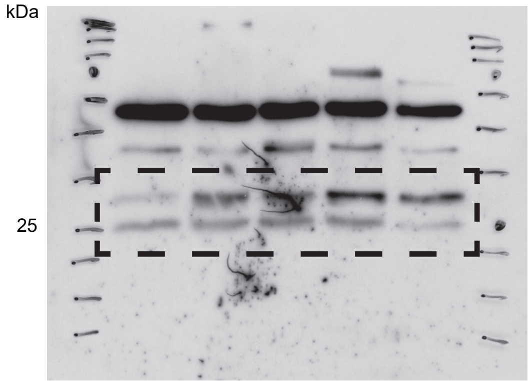

IB:VAPB-IP

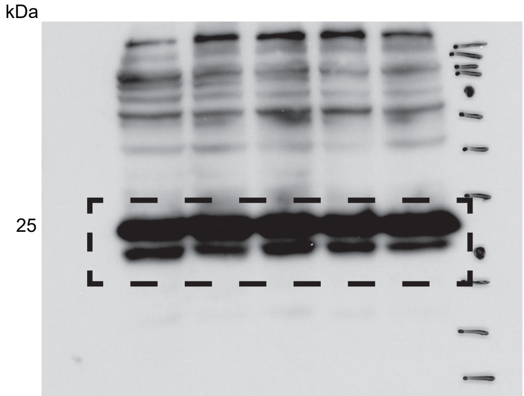

IB:VAPB-INPUT

11. Uncropped Western blots for Supplementary figure 6c

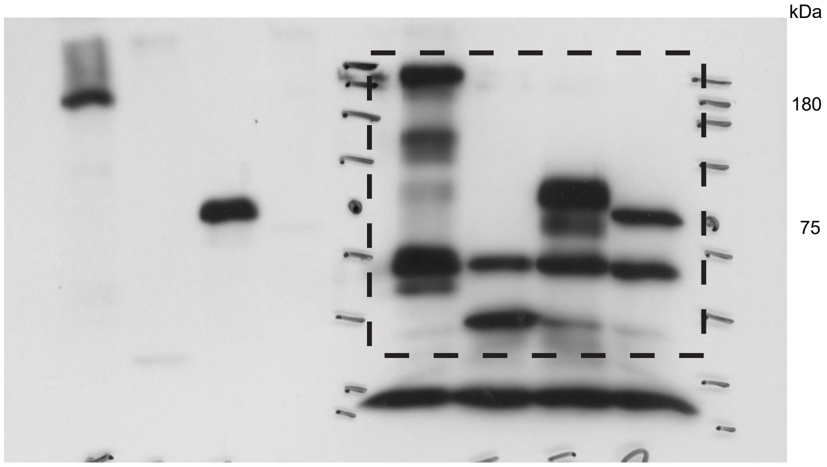

IB:HA-IP

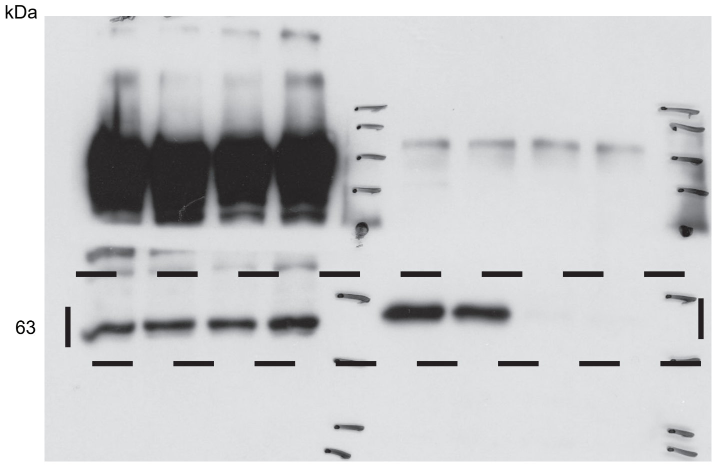

IB:DTNBP1-INPUT & IP

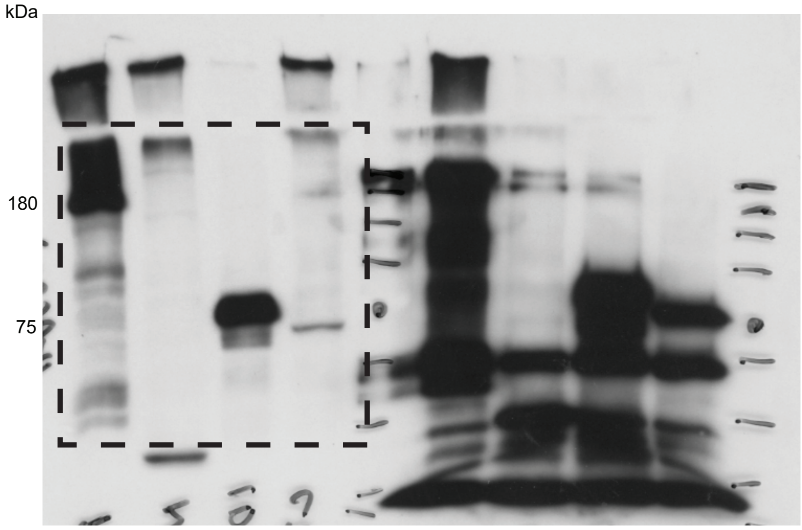

IB:HA-INPUT

12. Uncropped Western blots for Supplementary figure 6d

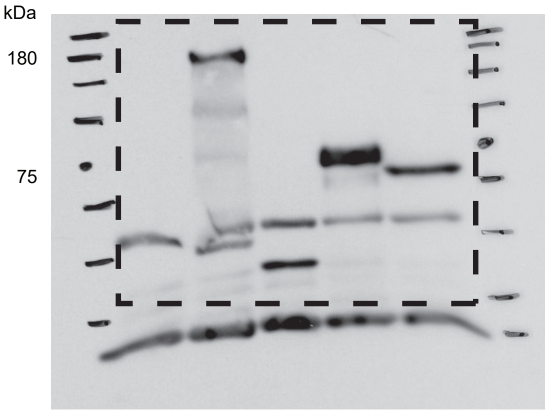

IB:HA-IP

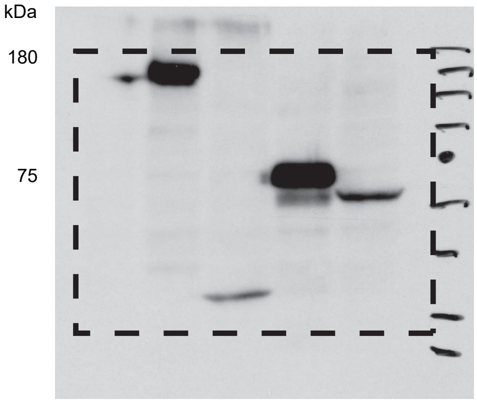

IB:HA-INPUT

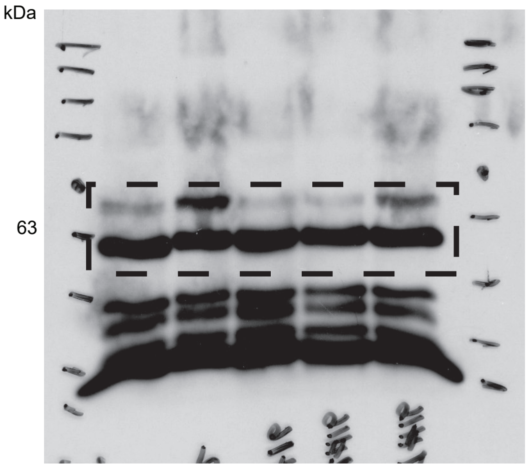

IB:SNX27-IP

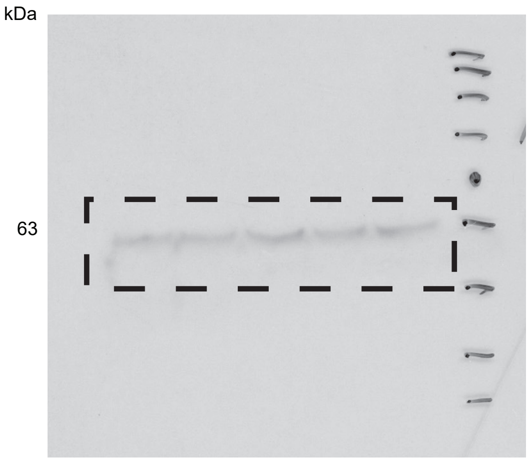

IB:SNX27-INPUT

13. Uncropped Western blots for Supplementary figure 6e

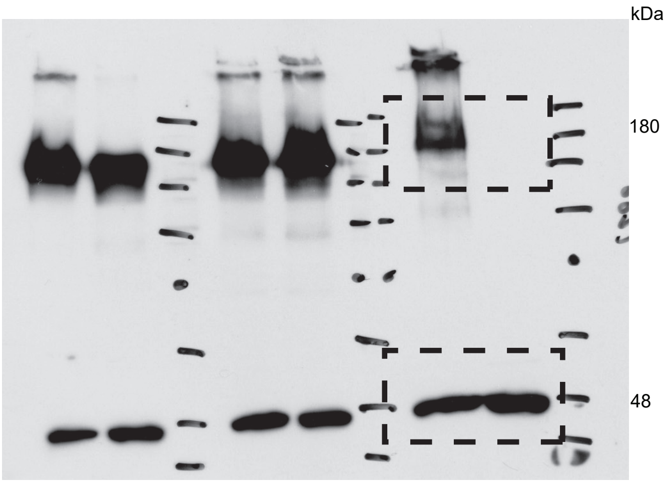

IB: TECPR2 & Actin

14. Uncropped Western blots for Supplementary figure 7f

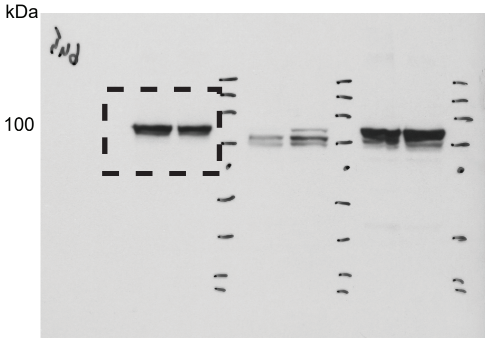

IB: VPS18

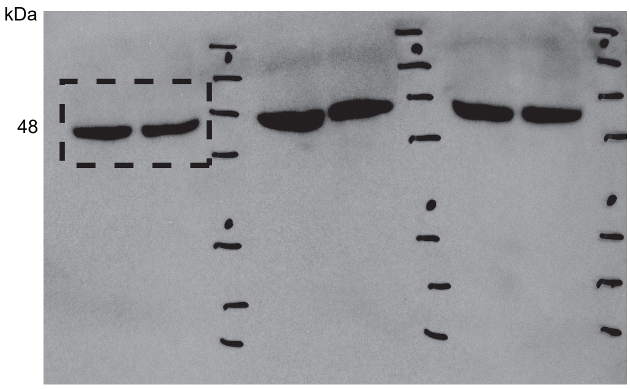

IB: Actin

15. Uncropped Western blots for Supplementary figure 7g

kDa
180

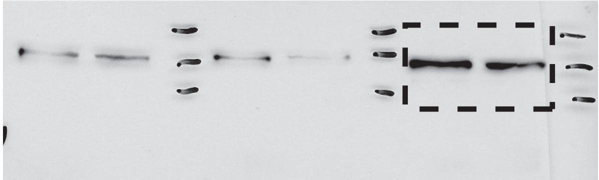

IB: EEA1

kDa
100

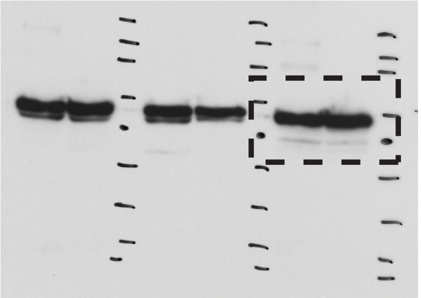

IB: Calnexin

16. Uncropped Western blots for Supplementary figure 7h

kDa
100

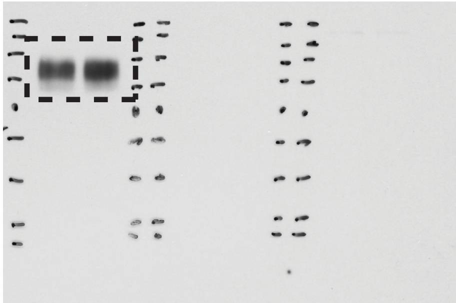

IB: LAMP1

kDa
48

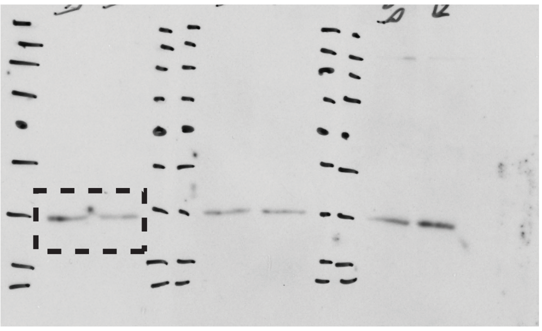

IB: Actin

17. Uncropped Western blots for Supplementary figure 7i

kDa
25

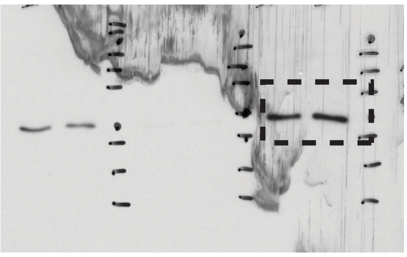

IB: RAB5

kDa
25

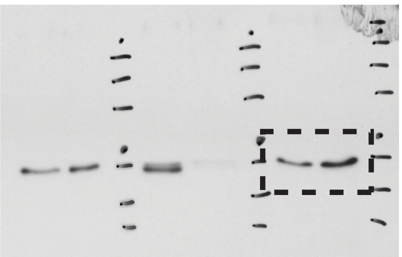

IB: RAB7

kDa
48

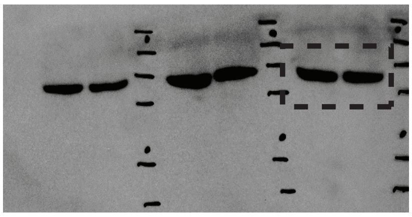

IB: Actin
